# Cell cycle and p53 gate the direct conversion of human fibroblasts to dopaminergic neurons

**DOI:** 10.1038/ncomms10100

**Published:** 2015-12-07

**Authors:** Houbo Jiang, Zhimin Xu, Ping Zhong, Yong Ren, Gaoyang Liang, Haley A. Schilling, Zihua Hu, Yi Zhang, Xiaomin Wang, Shengdi Chen, Zhen Yan, Jian Feng

**Affiliations:** 1 Veterans Affairs Western New York Healthcare System, Buffalo, New York 14215, USA; 2 Department of Physiology and Biophysics, State University of New York at Buffalo, Buffalo, New York 14214, USA; 3 Department of Neurology and Institute of Neurology, Ruijin Hospital Affiliated to Shanghai Jiao Tong University School of Medicine, Shanghai 200025, China; 4 Howard Hughes Medical Institute, Departments of Genetics & Pediatrics, Harvard Medical School, Boston, Massachusetts 02115, USA; 5 Center for Computational Research, New York State Center of Excellence in Bioinformatics & Life Sciences, State University of New York at Buffalo, Buffalo, New York 14260, USA; 6 Department of Neurobiology, Key Laboratory for Neurodegenerative Disorders of the Ministry of Education, Beijing Institute for Brain Disorders, Capital Medical University, Beijing 100069, China

## Abstract

The direct conversion of fibroblasts to induced dopaminergic (iDA) neurons and other cell types demonstrates the plasticity of cell fate. The low efficiency of these relatively fast conversions suggests that kinetic barriers exist to safeguard cell-type identity. Here we show that suppression of p53, in conjunction with cell cycle arrest at G1 and appropriate extracellular environment, markedly increase the efficiency in the transdifferentiation of human fibroblasts to iDA neurons by Ascl1, Nurr1, Lmx1a and miR124. The conversion is dependent on Tet1, as G1 arrest, p53 knockdown or expression of the reprogramming factors induces Tet1 synergistically. Tet1 knockdown abolishes the transdifferentiation while its overexpression enhances the conversion. The iDA neurons express markers for midbrain DA neurons and have active dopaminergic transmission. Our results suggest that overcoming these kinetic barriers may enable highly efficient epigenetic reprogramming in general and will generate patient-specific midbrain DA neurons for Parkinson’s disease research and therapy.

## Abstract (web summary)

The efficiency of reprogramming of fibroblasts into induced dopaminergic neurons is often low. Here, Jiang *et al.* demonstrate increased transdifferentiation rates by inducing cell cycle arrest, suppressing p53, Tet 1 and by adding neurotrophic factors to the media.

## Graphical abstract

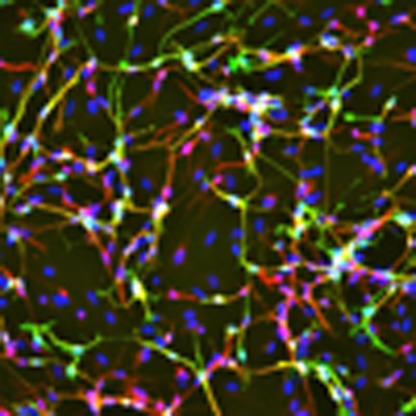

In a multicellular organism, different types of cells are generated in a deterministic manner during development from a single totipotent cell. This unidirectional process and the stability of cell-type identity suggest that a self-reinforcing mechanism is at work to maintain distinct cell-type identities, all of which are expressed from the same genome. A few earlier studies, for example, on MyoD^1^, have shown that cell-type identity can be radically changed by a single transcription factor, which rewires the transcription network in such a way that the same genome can express a different cell type. It has now been established that virtually any type of cell can be reprogrammed to induced pluripotent stem (iPS) cells by defined factors^2^ in a multistaged, stochastic process that requires many rounds of cell divisions^3^. Ablation of the p53 pathway greatly enhances the efficiency in the derivation of iPS cells^4^,^5^,^6^,^7^,^8^,^9^, although the underlying mechanism remains unclear. The plasticity of cell-type identity is further demonstrated by the direct conversion of fibroblasts to induced dopaminergic (iDA) neurons^10^,^11^,^12^ and other cell types^13^,^14^,^15^,^16^. Ascl1 is a critical transcription factor in the transdifferentiation of fibroblasts to induced neurons^17^. It converts embryonic carcinoma cells to neurons and causes rapid cell cycle exit, presumably by inducing the cyclin-dependent kinase inhibitor p27^Kip1^(ref. 18). Cellular reprogramming from one epigenetic state to another requires changes in epigenetic modifiers. Tet proteins are a family of three DNA hydroxylases that critically regulate the epigenome through the oxidation of 5-methylcytosine (5mC) to 5-hydroxymethylcytosine (5hmC) and other products^19^. The very high levels of 5hmC in neurons^20^ and the critical role of Tet proteins in epigenetic reprogramming, such as the derivation of iPSCs^21^, suggest that they may play important roles in epigenetic conversion of fibroblasts to neurons.

The direct conversion of fibroblasts to many types of cells, including iDA neurons, is a relatively fast process that manifests itself within days^10^,^11^,^12^. However, the low efficiency of the conversion suggests that conditions additional to the requisite transcription factors must be met to enable highly efficient cellular reprogramming. Identification of kinetic barriers to transdifferentiation would reveal significant mechanistic insight into cellular reprogramming in general and produce highly efficient ways to generate many types of useful cells from readily available sources such as fibroblasts. In this study, we show that attenuation of p53, in conjunction with cell cycle arrest at G1 and appropriate cell culture environment, increases the efficiency in the transdifferentiation of human fibroblasts to iDA neurons. The epigenetic conversion is dependent on Tet1, as p53 knockdown, G1 arrest and induction of reprogramming factors synergistically induce Tet1. Furthermore, Tet1 knockdown abolishes the conversion while its overexpression enhances the transdifferentiation. The iDA neurons express markers for midbrain DA neurons and exhibit active dopaminergic transmission.

## Results

### Attenuation of p53 increases the derivation of iDA neurons

**Figure 1:**
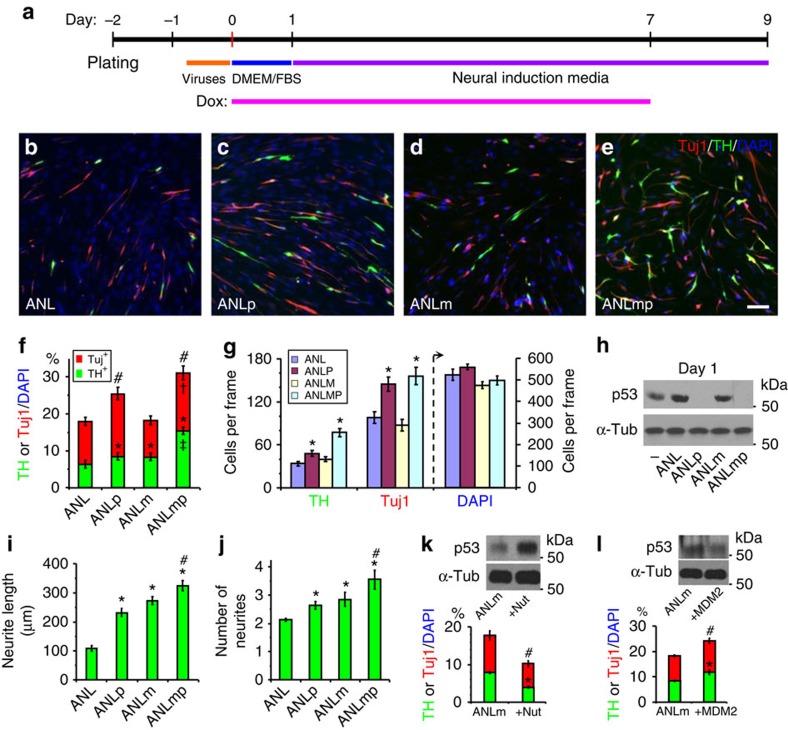
p53 attenuation enhances the conversion of human fibroblasts to iDA neurons. (**a**–**e**) Using the protocol in **a**, MRC5 cells were reprogrammed to iDA neurons in neural induction media with lentiviruses expressing Ascl1, Nurr1 and Lmx1a (ANL) (**b**), ANL plus p53 shRNA (ANLp) (**c**), ANL plus miR124 (ANLm) (**d**) or ANL plus miR124 and p53 shRNA (ANLmp) (**e**). Scale bar, 100 μm. (**f**,**g**) Percentage of TH^+^ or Tuj1^+^ cells in all cells (DAPI^+^) at day 9 (**f**). *^,#^*P*<0.05, unpaired, two-tailed Student’s *t*-tests versus TH^+^ or Tuj1^+^ in ANL, respectively. ^‡, †^*P*<0.05, unpaired, two tailed Student’s *t*-tests versus TH^+^ or Tuj1^+^ in ANLm, respectively; *n*=8 wells from four independent experiments for each condition. Average numbers of TH^+^, Tuj1^+^ or DAPI^+^ cells per frame under × 10 lenses were plotted in **g**. *P<0.05, unpaired, two tailed Student’s *t*-tests versus ANL for TH^+^ or Tuj1^+^ cells, respectively; *n*=8 wells from four independent experiments for each condition. The scale for DAPI^+^ cells is according to the *y* axis on the right. (**h**) Western blotting of p53 and α-tubulin (α-Tub) in total cell lysates at day 1. Total neurite length (**i**) and number of neurites (**j**) per TH^+^ neurons. *P<0.05, unpaired, two tailed Student’s *t*-tests versus ANL; ^#^*P*<0.05, unpaired, two tailed Student’s *t*-tests versus ANLm; *n*=30–60 TH^+^ neurons from three independent experiments for each condition. (**k**,**l**) The reprogramming efficiency with ANLm or ANLm plus nutlin-3a (**k**) or with ANLm plus MDM2 (**l**). *^, #^*P*<0.05, unpaired, two tailed Student’s *t*-tests versus TH^+^ or Tuj1^+^, respectively; *n*=6 wells from 3 independent experiments for each condition. Insets, western blotting of p53 and α-tubulin. Error bars denotes standard error of measurement.

Human fetal lung fibroblast MRC5 (Medical Research Council 5) cells, which are widely used in many reprogramming experiments^22^, were infected with tetracycline-inducible lentiviruses expressing human Ascl1, Nurr1 and Lmx1a (ANL)^10^, miRNA124 (refs 23, 24) and a constitutively active lentivirus for p53 shRNA. They were cultured in neural induction media^25^ with the protocol in Fig. 1a. After 3 days of induction with doxycycline (Dox, 1 μg ml^−1^), some cells exhibited a compact cell body with one or many long processes, reminiscent of neurons. When the cells were stained at day 9 with antibodies against tyrosine hydroxylase (TH), a marker for dopaminergic (DA) neurons, and β3-tubulin (Tuj1), a neuronal marker, we found that p53 knockdown significantly increased the generation of Tuj1^+^ cells (25.4±1.8% of all cells) and TH^+^ cells (8.4±0.8% of all cells) over the levels induced by ANL alone (17.9±1.2% for Tuj1^+^, 6.3±0.8 for TH^+^, *P*<0.05, unpaired, two-tailed Student’s *t*-tests; Fig. 1b,c,f). miR124 significantly enhanced the efficiency of ANL in generating TH^+^ cells (8.3±0.6%, *P*<0.05, unpaired, two-tailed Student’s *t*-tests), but not Tuj1^+^ cells (18.2±1.2%; Fig. 1d,f). The combination of ANL with miR124 and p53 shRNA (ANLmp) resulted in marked increases in Tuj1^+^ cells (31.1±1.9%, *P*<0.05, unpaired, two-tailed Student’s *t*-tests) and TH^+^ cells (15.4±1.1%, *P*<0.05, unpaired, two-tailed Student’s *t*-tests; Fig. 1e,f). The total number of cells (DAPI^+^) at day 9 was not significantly affected by the different combinations of viruses (Fig. 1g). Thus, the ratio of TH^+^/DAPI^+^ or Tuj1^+^/DAPI^+^ (that is, reprogramming efficiency) is consistent with the total number of TH^+^ or Tuj1^+^ cells (that is, reprogramming yield; Fig. 1g). p53 knockdown did not significantly change the total number of cells at day 9, as the initial overgrowth of mitotic cells led to more cell death after the reprogramming factors were turned on by DOX. Western blotting of p53 at day 1 confirmed its reduced expression by the p53 shRNA lentivirus, while miR124 did not have a significant effect on p53 level (Fig. 1h). Using NIH ImageJ with NeuronJ plugin^26^, we traced the neurites and analysed total neurite length and the number of neurites for TH^+^ cells derived from different conditions. p53 shRNA or miR124 significantly increased total neurite length (Fig. 1i) and the number of neurites (Fig. 1j), when compared with the situation with ANL alone. ANLmp produced even greater increases in total neurite length (Fig. 1i) and the number of neurites (Fig. 1j).

To substantiate the effect of p53 knockdown, we manipulated p53 levels using nutlin-3a, which inhibits MDM2-mediated degradation of p53 (ref. 27), or overexpression of MDM2 (ref. 28). Indeed, nutlin-3a increased p53 level and significantly decreased reprogramming efficiency (Fig. 1k). Conversely, overexpression of MDM2 reduced p53 level and significantly increased reprogramming efficiency (Fig. 1l). We performed array comparative genomic hybridization to compare genomic DNA from MRC5 fibroblasts and MRC5-derived induced neurons. There was no significant genomic change (Supplementary Fig. 1), which is consistent with the very low expression level of the p53 shRNA transgene (Supplementary Fig. 2). All the other four transgenes (ANLm) were greatly silenced in induced neurons (Supplementary Fig. 2).

### The effect of p53 knockdown is not affected by p21

**Figure 2:**
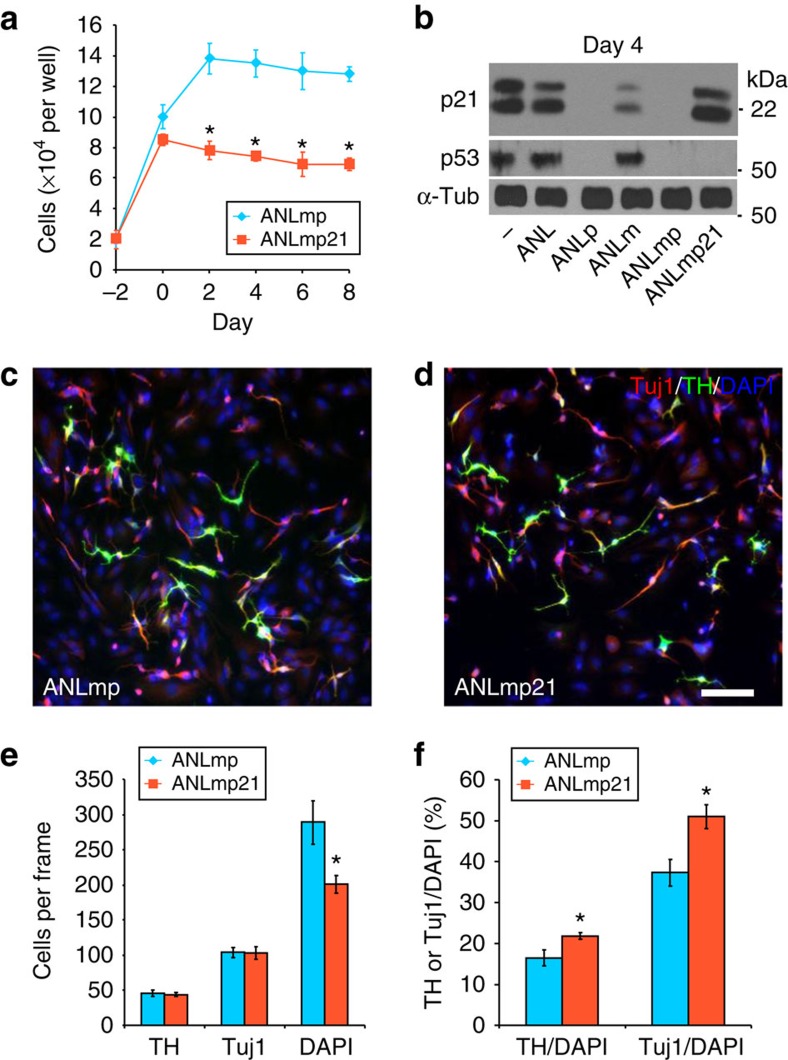
The effect of p53 knockdown is not affected by p21. (**a**) The number of live cells by trypan blue staining at different days for MRC5 cells transduced with lentiviruses expressing ANLmp or ANLmp plus p21 (ANLmp21). (**b**) Western blotting of p21, p53 and α-tubulin (α-Tub) in total cell lysates from MRC5 cells transduced with the indicated lentiviruses at day 4. (**c**,**d**) Representative images of iDA neurons converted from MRC5 cells transduced with lentiviruses expressing ANLmp (**c**) or ANLmp21 (**d**) in neural induction media. Scale bar, 100 μm. (**e**,**f**) Quantification of the number of TH^+^, Tuj1^+^ or DAPI^+^ cells at day 9 (**e**), and the ratio of TH^+^/DAPI^+^ or Tuj1^+^/DAPI^+^ at day 9 (**f**). *P<0.05, unpaired, two tailed Student’s *t*-tests versus ANLmp for the same category; *n*=6 wells from 3 independent experiments for each condition.

p53 induces p21, which binds to and inhibits cyclin-dependent kinases (CDKs), preventing the phosphorylation of critical CDK substrates and thus blocking cell cycle progression^29^. Consequently, p53 knockdown promotes cell proliferation in mitotic cells. To investigate whether the enhancing effect of p53 knockdown on the derivation of iDA neurons is connected to the effect of p53 knockdown on cell cycle progression, we tested whether overexpression of p21, which blocks the proliferative effect of p53 knockdown, affects derivation of iDA neurons. The same number of MRC5 cells (2 × 10^4^ per well) were plated on day −2 and infected on day −1 with lentiviruses expressing ANLmp or ANLmp plus p21 (ANLmp21) according to the protocol in Fig. 1a. Dox was added at day 0 to induce the expression of ANLm, while the expression of p53 and p21 were constitutive. We trypsinized the cells at different days to count the total number of live cells by trypan blue staining. Overexpression of p21 led to significantly fewer cells in comparison to ANLmp alone (*P*<0.05, unpaired, two-tailed Student’s *t*-tests; Fig. 2a), as cell death was visibly increased. p53 knockdown ablated the expression of p53 and p21. Overexpression of exogenous p21 restored p21 level (Fig. 2b). The double bands for p21 is due to its phosphorylation^30^. Full western blotting images of p53 and p21 are shown in Supplementary Fig. 3. There was no significant difference in the number of TH^+^ or Tuj1^+^ cells when the cultures were stained at day 9 (*P*>0.05, unpaired, two-tailed Student’s *t*-tests). However, the number of DAPI^+^ cells was significantly reduced (*P*<0.05, unpaired, two-tailed Student’s *t*-tests, ANLmp21 versus ANLmp; Fig. 2c–e). It seems that p21 overexpression and p53 knockdown caused a conflict only in mitotic cells, which decreased the number of these cells (DAPI^+^/Tuj1^−^, Fig. 2e) through reduced proliferation and increased cell death. Thus, the percentage of TH^+^ or Tuj1^+^ cells in DAPI^+^ cells was increased significantly (*P*<0.05, unpaired, two-tailed Student’s *t*-tests, ANLmp21 versus ANLmp; Fig. 2f). The results suggest that the effect of p53 knockdown on the reprogramming of fibroblasts to iDA neurons is independent of the effect of p53 on cell cycle. The two effects are actually opposing, as p53 knockdown increases cell proliferation while the fibroblast-to-neuron conversion generates postmitotic neurons. Thus, the conversion efficiencies as measured by TH^+^/DAPI^+^ or Tuj1^+^/DAPI^+^ ratios were increased when p21 blocked the proliferative effect of p53 knockdown.

### G1 arrest facilitates the conversion to iDA neurons

**Figure 3:**
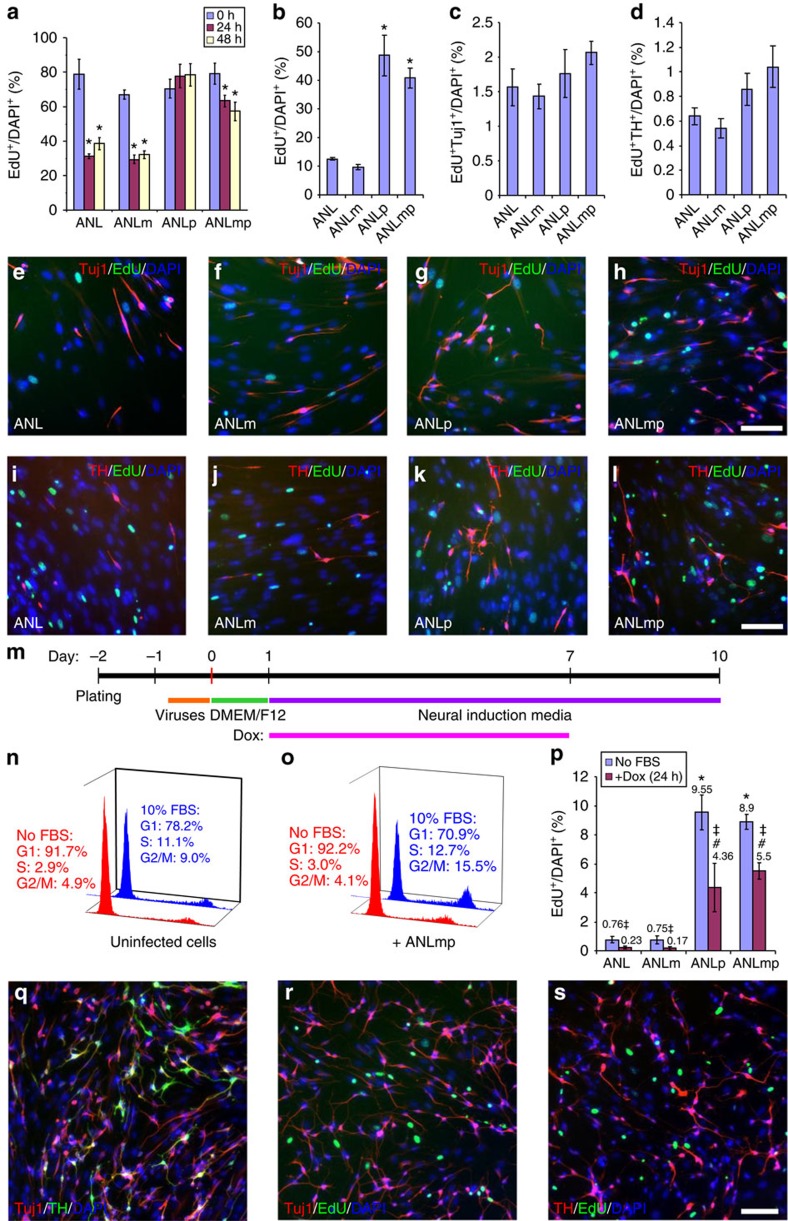
G1 arrest facilitates fibroblast-to-iDA conversion. (**a**) The percentage of EdU-labelled cells after Dox induction of the indicated reprogramming factors for 0, 24 and 48 h. *P<0.01, unpaired, two tailed Student’s *t*-tests versus 0 h; *n*=6 wells from 3 independent experiments for each condition. (**b**–**d**) The percentage of EdU^+^ cells in all DAPI^+^ cells (**b**), the percentage of EdU^+^Tuj1^+^ double-positive cells in all DAPI^+^ cells (**c**) and the percentage of EdU^+^TH^+^ double-positive cells in all DAPI^+^ cells (**d**) at day 9 of reprogramming with the indicated factors. *P<0.05, unpaired, two tailed Student’s *t*-tests versus ANL or ANLm; *n*=6 wells from 3 independent experiments. Representative images of cells co-stained for Tuj1/EdU/DAPI (**e**–**h**) or TH/EdU/DAPI (**i**–**l**) at day 9 of reprogramming with the indicated factors. (**m**) Improved protocol using serum withdrawal for 24 h in DMEM/F12 to arrest cell cycle at G1 before Dox induction of reprogramming factors in neural induction media. Cell cycle analysis for MRC5 cells (**n**) or MRC5 cells transduced with ANLmp viruses (**o**) in the presence or absence of fetal bovine serum (FBS). (**p**) Percentage of EdU-labelled cells after serum withdrawal and treatment without or with Dox for 24 h for the indicated reprogramming factors. ^**‡**^*P*<0.05, unpaired, two tailed Student’s *t*-tests versus the preceding bar. *^, #^*P*<0.05, unpaired, two tailed Student’s *t*-tests versus ANL without or with Dox, respectively; *n*=6 wells from 3 independent experiments. (**q**) TH^+^ and Tuj1^+^ neurons generated with the protocol in **m**. Lack of EdU labelling in Tuj1^+^ neurons (**r**) and TH^+^ neurons (**s**) derived from the protocol in **m**. Pulse labelling of EdU (for 2 h) was performed at 24 h in Dox treatment. Staining was done at day 10. Scale bars, 100 μm.

The dual effects of p53 knockdown in enhancing fibroblast-to-neuron conversion and cell proliferation led us to examine how cell cycle affects the transdifferentiation. We found that p53 knockdown (in ANLp or ANLmp) markedly increased the percentage of 5-ethynyl-2′-deoxyuridine-positive (EdU^+^) cells (Fig. 3a and Supplementary Fig. 4). In contrast, the induction of ANL (or ANLm) by Dox rapidly and markedly reduced the percentage of EdU^+^ cells, suggesting that the conversion of fibroblasts to neurons is accompanied by rapid cell cycle exit (within 24 h of Dox induction), as have been reported previously^13^,^18^. Fibroblasts infected with ANL, ANLm, ANLp or ANLmp were pulse labelled with EdU for 2 at 24 h after Dox induction and were cultured to the completion of reprogramming. At day 9, the percentage of EdU^+^ cells, which were in S phase at 24 h after DOX induction, was significantly increased by p53 knockdown in ANLp or ANLmp, compared with ANL or ANLm (*P*<0.05, unpaired, two-tailed Student’s *t*-tests; Fig. 3b). However, only about 1.5–2% of all cells were double positive for Tuj1 and EdU (Fig. 3c,e–h) and about 0.5–1% of all cells were double positive for TH and EdU (Fig. 3d,i–l), no matter what combinations of viruses were used (*P*>0.05). The vast majority of EdU^+^ cells did not become Tuj1^+^ or TH^+^ neurons.

These findings made us realize that we need to reduce the percentage of cells in S phase to promote reprogramming. This could be easily achieved by culturing virus-infected cells in Dulbecco’s modified eagle medium (DMEM)/F12 media without serum for 24 h (Fig. 3m). After serum withdrawal, uninfected (Fig. 3n) or ANLmp-infected fibroblasts (Fig. 3o) were arrested at the G1 phase, with significantly fewer percentages of cells in S and G2/M phases. Accordingly, the percentage of EdU^+^ cells at this condition was lowered to 8.9±0.7% (Fig. 3p and Supplementary Fig. 5d). Dox induction for 24 h further decreased the percentage of EdU^+^ cells to 5.5±0.4% (Fig. 3p and Supplementary Fig. 5h). Serum withdrawal and Dox induction of ANL or ANLm achieved greater reduction in the percentage of EdU^+^ cells, as p53 knockdown in ANLp or ANLmp promoted more cells to enter the S phase (Fig. 3p and Supplementary Fig. 5). With cell cycle arrest at G1 induced by serum withdrawal, ANLmp produced markedly more Tuj1^+^ cells and TH^+^ cells at day 10 (Fig. 3q). None of these neurons was EdU^+^ (Fig. 3r,s), as the percentage of S phase cells was already reduced markedly by serum withdrawal at the start of reprogramming.

### G1 arrest by different methods all enhances the conversion

**Figure 4:**
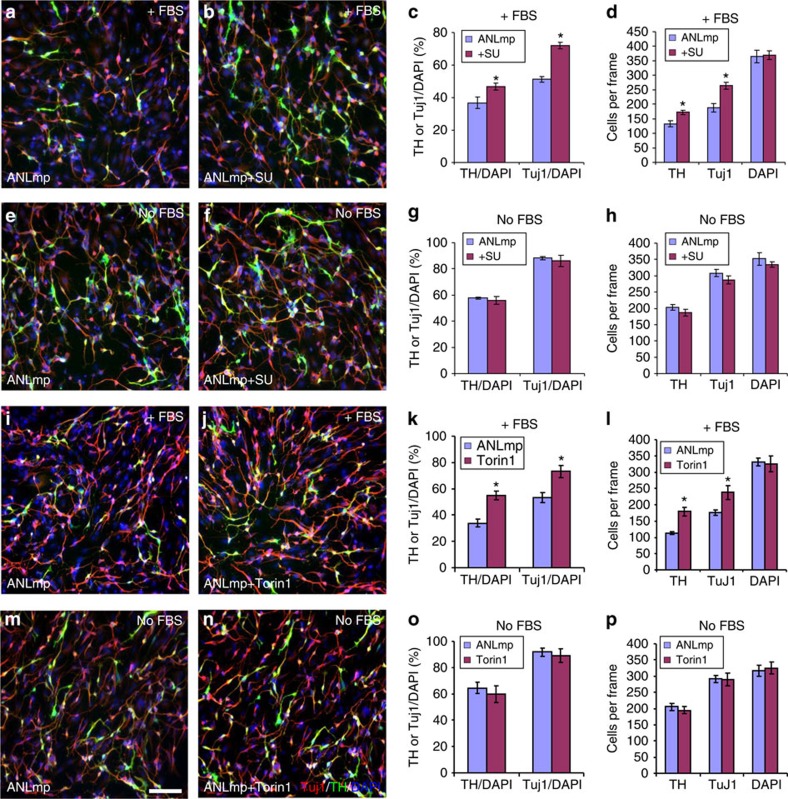
G1 arrest by different mechanisms enhances the transdifferentiation. (**a**–**d**) Representative images of MRC5 cells reprogrammed in full media by ANLmp in the presence of fetal bovine serum (FBS) without (**a**) or with the CDK2 inhibitor SU9516 (SU, 5 μM for 48 h) (**b**). The percentage of TH^+^ or Tuj1^+^ cells in all cells (DAPI^+^) (**c**) and the number of TH^+^, Tuj1^+^ or DAPI^+^ cells per frame under × 10 lenses (**d**) were analysed at day 10. *P<0.05, unpaired, two tailed Student’s *t*-tests versus the preceding bar; *n*=6 wells from 3 independent experiments. (**e**–**h**) Representative images of MRC5 cells reprogrammed in full media by ANLmp in the absence of FBS without (**e**) or with the CDK inhibitor SU9516 (SU, 5 μM for 48 h) (**f**). The percentage of TH^+^ or Tuj1^+^ cells in all cells (DAPI^+^) (**g**) and the number of TH^+^, Tuj1^+^ or DAPI^+^ cells per frame under × 10 lenses (**h**) were analysed at day 10. (**i**–**l**) Representative images of MRC5 cells reprogrammed in full media by ANLmp in the presence of FBS without (**i**) or with the mTOR inhibitor Torin 1 (0.1 μM for 48 h) (**j**). The percentage of TH^+^ or Tuj1^+^ cells in all cells (DAPI^+^) (**k**) and the number of TH^+^, Tuj1^+^ or DAPI^+^ cells per frame under × 10 lenses (**l**) were analysed at day 10. *P<0.05, unpaired, two tailed Student’s *t*-tests versus the preceding bar; *n*=6 wells from 3 independent experiments. Representative images of MRC5 cells reprogrammed in full media by ANLmp in the absence of FBS without (**m**) or with the mTOR inhibitor Torin 1 (0.1 μM for 48 h) (**n**). The percentage of TH^+^ or Tuj1^+^ cells in all cells (DAPI^+^) (**o**) and the number of TH^+^, Tuj1^+^ or DAPI^+^ cells per frame under × 10 lenses (**p**) were analysed at day 10. Scale bar, 100 μm.

To substantiate the effect of G1 arrest on the transdifferentiation, we cultured ANLmp-infected MRC5 cells for 24 h in 10% fetal bovine serum (FBS) or serum plus SU9516 (5 μM), a selective inhibitor of CDK2 (ref. 31). We then added Dox to initiate reprogramming in full media (see below) without or with SU9516 for another 24 h. SU9516 significantly increased reprogramming efficiency (Fig. 4c) and yield (Fig. 4d), compared with the situation with 10% FBS alone (Fig. 4a–d). The number of DAPI^+^ cells was not significantly affected by 48 h treatment of SU9516 (Fig. 4d), which did not significantly increase cell death during the course of reprogramming. This is in sharp contrast to the constitutive expression of p21, which greatly reduced DAPI^+^ non-neural cells through increased cell death (Fig. 2a,e). When we performed the same experiments with serum withdrawal during the 24 h before Dox addition, we found that SU9516 did not further increase the conversion of fibroblasts to iDA neurons (Fig. 4e–h). The effect of SU9516 was occluded by serum withdrawal.

Furthermore, we used Torin1, a highly selective mTOR inhibitor to arrest cell cycle at the G1/S checkpoint^32^,^33^. ANLmp-infected MRC5 cells were treated without or with Torin1 (0.1 μM) for 24 h in 10% FBS. Dox was then added to initiate reprogramming in full media (see below) without or with Torin1 for another 24 h. Torin1 significantly increased reprogramming efficiency (Fig. 4k) and yield (Fig. 4l), compared with the situation with 10% FBS alone (Fig. 4i–l). The total number of DAPI^+^ cells was not significantly changed by the Torin1 treatment (Fig. 4l). The same experiments were repeated with serum withdrawal during the 24 h before the addition of Dox. The transdifferentiation-enhancing effect of Torin1 was occluded by serum withdrawal (Fig. 4m–p). Thus, G1 arrest induced by three different methods—serum withdrawal, the CDK2 inhibitor SU9516 or the mTOR inhibitor Torin1—all enhanced the conversion of fibroblasts to iDA neurons.

To examine the effects of cell quiescence, ANLmp-infected MRC5 cells were maintained for 4 days in DMEM media with 0.1% FBS to induce quiescence^34^,^35^. Dox was then added to initiate reprogramming in full media. After another 9 days, cells were fixed and stained for Tuj1, TH and DAPI (Supplementary Fig. 6a,b). Reprogramming efficiency (Supplementary Fig. 6c) and yield (Supplementary Fig. 6d) were significantly decreased when quiescent, rather than G1-arrested MRC5 cells, were used (*P*<0.05, unpaired, two-tailed Student’s *t*-tests). This is consistent with studies showing that quiescent fibroblasts are much harder to reprogram by MyoD to myocytes^34^,^35^. To examine the impact of cell senescence, we converted MRC5 cells at passage 1 or passage 10 to iDA neurons (Supplementary Fig. 6e,f) and found that reprogramming efficiency (Supplementary Fig. 6g) and yield (Supplementary Fig. 6h) were significantly reduced by cellular senescence, which manifested in the significantly slower cell proliferation rate of MRC5 cells at passage 10 versus that at passage 1 (Supplementary Fig. 6i; *P*<0.05, unpaired, two-tailed Student’s *t*-tests).

### Synergistic actions of media additives on the conversion

**Figure 5:**
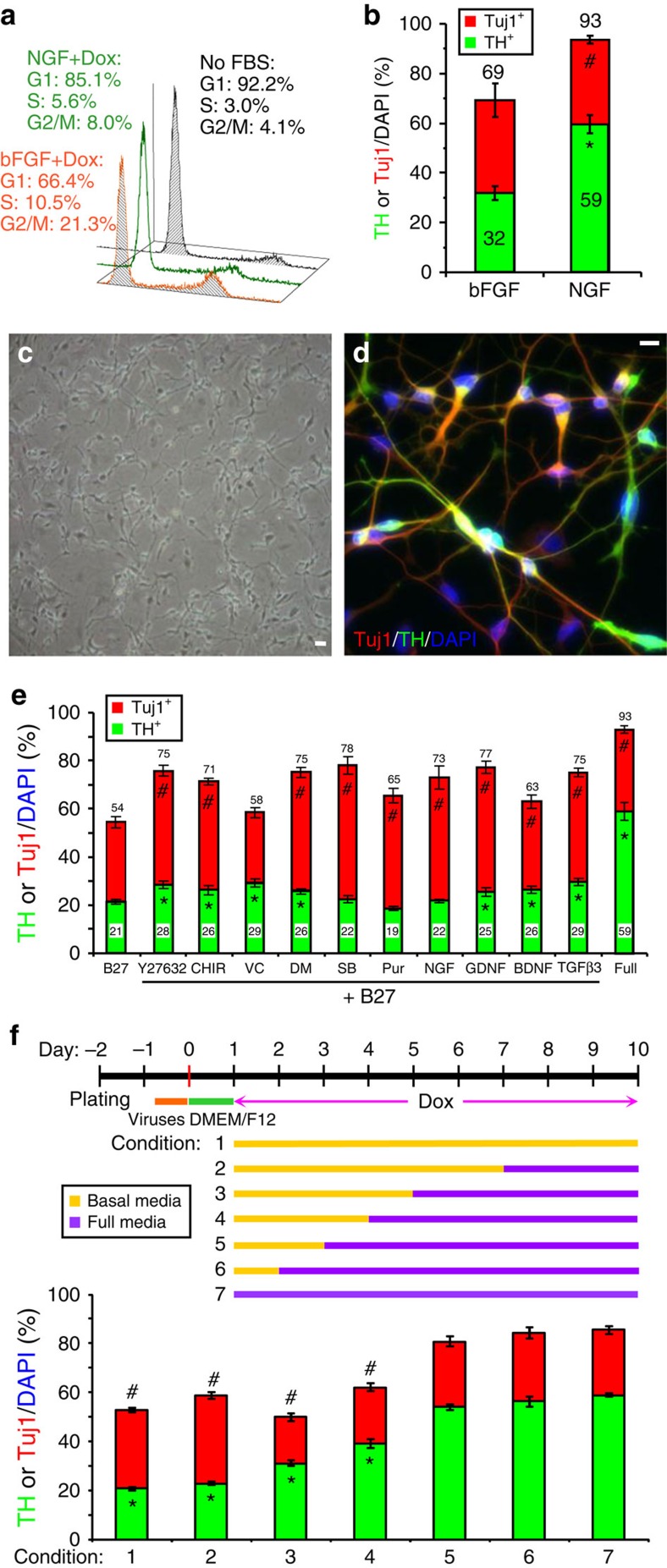
Neurotrophic factors and small-molecule compounds augment the conversion. (**a**) ANLmp-transduced MRC5 cells were cultured in the absence of serum for 24 h (black line) and treated with NGF and Dox (green line) or bFGF and Dox (red line) for another 24 h. Cell cycle analysis by propidium iodide staining was performed on these cells. (**b**–**d**) The percentage of TH^+^ or Tuj1^+^ neurons at day 10 in neural induction media containing bFGF or NGF (that is, full media) (**b**) *^, #^*P*<0.01, unpaired, two tailed Student’s *t*-tests versus TH^+^ or Tuj1^+^ in bFGF, respectively; *n*=6 wells from 3 independent experiments. Representative phase contrast image (**c**) and immunostaining (**d**) of neurons generated in full media containing NGF. Scale bars, 10 μm. (**e**) The percentage of TH^+^ or Tuj1^+^ neurons at day 10 in basal media without or with various small molecule compounds and neurotrophic factors. B27, B27 supplements; VC, vitamin C; DM, dorsomorphin; SB, SB431542; CHIR, CHIR99021; Pur, Purmorphamine; NGF, nerve growth factor; GDNF, glial cell line-derived neurotrophic factor; BDNF, brain-derived neurotrophic factor; TGFβ3, transforming growth factor β3; Y27632, rock inhibitor; Full, all of the above. *^, #^*P*<0.05, unpaired, two tailed Student’s *t*-tests versus TH^+^ or Tuj1^+^ in B27 alone, respectively; *n*=6 wells from 3 independent experiments for each condition. (**f**) The percentage of TH^+^ or Tuj1^+^ neurons at day 10 when basal media and full media were used at different time as indicated. *^, #^*P*<0.05, unpaired, two tailed Student’s *t*-tests versus TH^+^ or Tuj1^+^ for condition 7, respectively; *n*=6 wells from 3 independent experiments for each condition.

In our initial reprogramming experiments, we used neural induction media^25^ that contained basic fibroblast growth factor (bFGF, 20 ng ml^−1^) and many other small-molecule compounds and growth factors (see Methods). When we performed cell cycle analysis on ANLmp-infected fibroblasts that were treated with Dox in this media for 24 h, we found that bFGF in neural induction media promoted cell cycle entry in the absence of serum (Fig. 5a). By replacing bFGF with nerve growth factor (NGF, 20 ng ml^−1^), we found that the new media (termed full media) maintained cell cycle arrest at G1 (Fig. 5a) and significantly increased the efficiency in the conversion of human fibroblasts to neurons (*P*<0.05, unpaired, two-tailed Student’s *t*-tests). At day 10 in full media with NGF, 93.3±1.6% of all cells were Tuj1^+^ and 59.2±3.7% of all cells were TH^+^, while the percentage of Tuj1^+^ or TH^+^ cells was 68.9±6.7% or 31.7±2.8%, respectively, in the same media with bFGF instead of NGF (Fig. 5b). Representative phase contrast image (Fig. 5c) and immunostaining (Fig. 5d) showed that reprogramming with full media produced a very high percentage of neurons, with many of them expressing TH. We found that Dox induction from day 1 to day 7 achieved the best results (Supplementary Fig. 7a,b). The rapid pace of reprogramming is shown in Supplementary Fig. 7d–i.

The different effects of bFGF and NGF led us to systematically examine the impact of various additives in full media. Comparing with the situation using basal media (DMEM/F12 with B27 and N2 supplements), reprogramming efficiency was improved by the singular addition of the ROCK inhibitor Y27632 (10 μM)^36^, CHIR99021 (CHIR, 3 μM)^37^, vitamin C (VC, 0.2 mM)^38^, dorsomorphin (DM, 1 μM)^39^, SB431542 (SB, 10 μM)^40^, purmorphamine (Pur, 2 μM)^41^, NGF (20 ng ml^−1^), glial cell line-derived neurotrophic factor (GDNF; 20 ng ml^−1^)^25^, brain-derived neurotrophic factor (BDNF; 20 ng ml^−1^)^25^ or transforming growth factor (TGF)β3 (1 ng ml^−1^)^25^. However, the most remarkable improvement was found when all these factors were included in the full media (Fig. 5e). Representative images and reprogramming yields were shown in Supplementary Fig. 8a–o. TGFβ3 also activates the p38 MAP kinase pathway^42^ to support the differentiation of midbrain DA neurons^43^. At 10 μM, SB431542 only partially inhibits TGFβ signalling in the differentiation of midbrain DA neurons^44^, as TGFβ3 can bind to type II TGF receptors, without the need for type I receptors^45^, which SB431542 blocks^46^.

To explore whether additives in full media facilitate the conversion *per se* or enhance the survival of converted cells, we used basal media and full media at different time points. As shown in Fig. 5f, there was no significant difference in reprogramming efficiency when the first 1 or 2 days of Dox treatment were in basal media (condition 6 or 5, respectively) or in full media (condition 7), as long as the later days were in full media. Prolonged incubation of the cells (3 days or more) in basal media significantly decreased reprogramming efficiency (*P*<0.05, unpaired, two-tailed Student’s *t*-tests). Reprogramming yields were consistent with reprogramming efficiency, as the total numbers of cells (DAPI^+^) were not significantly affected by the seven conditions (*P*>0.05, unpaired, two-tailed Student’s *t*-tests; Supplementary Fig. 8p–r). By day 3, most of the cells already assumed neuronal morphology (Supplementary Fig. 7f). The rapid pace of conversion suggests that an intrinsic programme is being executed during the first 2 days of Dox treatment. The lack of effects of the media additives during the rapid conversion suggests that they improve reprogramming largely by promoting survival of converted cells.

With or without p53 knockdown, serum withdrawal increased reprogramming efficiency for all combinations of reprogramming factors (ANL, ANLp, ANLm and ANLmp; Supplementary Fig. 9a–h versus Fig. 1b–f), suggesting that the transdifferentiation-enhancing effects exerted by G1 arrest and p53 knockdown are independent. To substantiate the results from MRC5 cells (fetal, male; Supplementary Fig. 9h), we reprogrammed additional lines of normal human primary fibroblasts including IMR90 (fetal, female; Supplementary Fig. 9i), CCD-19 LU (20 years, female; Supplementary Fig. 9j), AG22056 (newborn, male; Supplementary Fig. 9k,n), AG16146 (31 years, male; Supplementary Fig. 9l,n) and GM00731 (96 years, male; Supplementary Fig. 9m,n), using ANLmp and serum withdrawal. Fetal fibroblasts (MRC5 and IMR90) behaved the same way (Supplementary Fig. 9h,i), while fibroblasts from adults (CCD-19Lu, AG16146 and GM00731) and the newborn (AG22056) showed reduced conversion efficiency (Supplementary Fig. 9j–n), as has been similarly found in other studies^10^,^11^. The effect of miR124 on enhancing the morphology of iDA neurons (Fig. 1b–e,i,j) became more apparent when conversion was done without serum (Supplementary Fig. 9a versus c or Supplementary Fig. 9b versus d). In addition, miR124 significantly increased the efficiency of conversion (Fig. 1f for cells in serum) and much more so with serum withdrawal (Supplementary Fig. 9h–j; *P*<0.05, unpaired, two-tailed Student’s *t*-tests). Individually, the effect of miR124, p53shRNA, G1 arrest or media additives was relatively small. However, they were additive so that the accumulative effects were very substantial, raising the efficiency 9.4-fold for TH^+^ cells (Supplementary Fig. 10a) and 5.2-fold for Tuj1^+^ cells (Supplementary Fig. 10b). The effect of p53 knockdown on the conversion was substantiated by co-expressing p53 shRNA and green fluorescent protein (GFP) in a bicistronic vector (pLKO.3G). The percentage of Tuj1^+^ cells in GFP^+^ cells was much higher than that in GFP^−^ cells (Supplementary Fig. 10c). The same was true for the percentage of TH^+^ cells (Supplementary Fig. 10d). The best time to freeze the cells was at 24 h of serum withdrawal. ANLmp-infected MRC5 cells were cultured for 24 h without serum and then frozen for 7 days. After cells were thawed, Dox was added to reprogram the cells in full media for another 9 days. Conversion efficiency and yield were comparable to the situation where cells were reprogrammed without freezing (Supplementary Fig. 11). Once reprogramming was initiated by Dox, freezing the cells at any point caused lots of cell death.

### The conversion of fibroblasts to iDA neurons depends on Tet1

**Figure 6:**
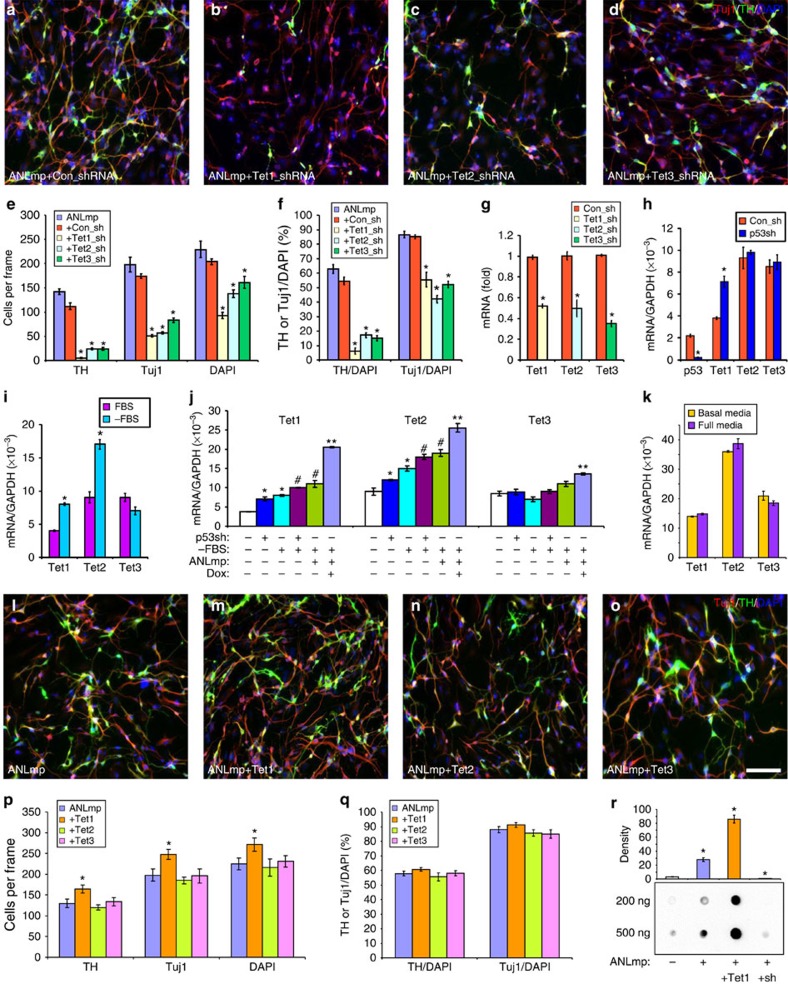
The conversion is dependent on Tet1. (**a**–**d**) MRC5 cells reprogrammed with ANLmp and shRNA against scrambled sequence (Con_shRNA) (**a**), Tet1 (**b**), Tet2 (**c**) or Tet3 (**d**) were stained for Tuj1, TH and DAPI. (**e**, **f**) Reprogramming yield (**e**) and efficiency (**f**). *P<0.05, unpaired, two tailed Student’s *t*-tests versus ANLmp; *n*=6 wells from 3 independent experiments. (**g**) mRNA levels of Tet genes in MRC5 cells transduced with shRNA against control sequence, Tet1, Tet2 or Tet3. *P<0.05, unpaired, two tailed Student’s *t*-tests versus the preceding bar; *n*=6. (**h**) mRNA levels of p53 and Tet genes in MRC5 cells transduced with control shRNA or p53 shRNA. *P<0.05, unpaired, two-tailed Student’s *t*-tests versus the preceding bar; *n*=6. (**i**) mRNA levels of Tet genes in MRC5 cells with or without 10% FBS for 24 h. *P<0.05, unpaired, two-tailed Student’s *t*-tests versus the preceding bar; *n*=6. (**j**) MRC5 cells were treated as indicated to measure mRNA levels of Tet genes. *P<0.05 versus the first bar for each Tet gene; ^#^*P*<0.05 versus the second or third bar for each Tet gene; **P<0.05 versus all other bars for each Tet gene; *n*=6, all with unpaired, two-tailed Student’s *t*-tests. (**k**) mRNA levels of Tet genes in ANLmp-infected MRC5 cells reprogrammed in basal media or full media for 2 days; *n*=6. (**l**-**o**) MRC5 cells reprogrammed with ANLmp (**l**) or ANLmp plus Tet1 (**m**), Tet2 (**n**) or Tet3 (**o**) were stained for Tuj1, TH and DAPI. (**p**, **q**) Reprogramming yield (**p**) and efficiency (**q**). *P<0.05, unpaired, two-tailed Student’s *t*-tests versus ANLmp for the same category; *n*=6. (**r**) 5hmC dot blot of genomic DNA from MRC5 cells reprogrammed as indicated for 2 days. Blots from 200 ng DNA were quantified. *P<0.05, unpaired, two-tailed Student’s *t*-tests versus ANLmp alone; *n*=3 independent experiments. Scale bar, 100 μm.

The very high levels of 5hmC in neurons^20^ and the critical role of Tet proteins in epigenetic reprogramming, such as the derivation of iPSCs^21^, led us to explore the potential role of Tet proteins in the direct conversion of fibroblasts to iDA neurons. MRC5 cells were reprogrammed with lentiviruses expressing ANLmp and shRNA against scrambled control sequence (Fig. 6a), Tet1 (Fig. 6b), Tet2 (Fig. 6c) or Tet3 (Fig. 6d). Knocking down each Tet, particularly Tet1, greatly reduced the numbers of TH^+^ cells, Tuj1^+^ cells and DAPI^+^ cells (Fig. 6e), due to large amounts of cell death. The percentages of TH^+^ or Tuj1^+^ cells in DAPI^+^ cells were also severely decreased, when compared with those produced by ANLmp alone or ANLmp plus scrambled shRNA (Fig. 6f). Real-time quantitative reverse transcription–PCR (qRT–PCR) measurement of Tet1, Tet2 or Tet3 level in MRC5 cells transduced with lentivirus expressing shRNA against scrambled sequence, Tet1, Tet2 or Tet3 for 3 days showed that each of the Tet genes was significantly knocked down (Fig. 6g).

As p53 knockdown significantly enhanced the conversion of fibroblasts to iDA neurons (Fig. 1), we measured the level of Tet1, Tet2 or Tet3 by qRT–PCR in MRC5 cells transduced with p53 shRNA for 1 day. The mRNA level of p53 was greatly reduced by the p53 shRNA lentivirus, while the expression of Tet1, but not Tet2 or Tet3, was significantly increased (*P*<0.05, unpaired, two-tailed Student’s *t*-tests; Fig. 6h). Next, we examined the impact of serum withdrawal on Tet expression and found that the mRNA levels of Tet1 and Tet2, but not Tet3, were significantly increased in MRC5 cells cultured in the absence of serum for 24 h (*P*<0.05, unpaired, two-tailed Student’s *t*-tests; Fig. 6i). When MRC5 cells were transduced with p53 shRNA and cultured without serum for 24 h, there was an even higher induction of Tet1 and Tet2, but not Tet3 (Fig. 6j). Infection of ANLmp lentiviruses did not significantly induce Tet genes further (Fig. 6j). However, Dox-induced expression of ANLm for 2 days (p53 shRNA was constitutively expressed) led to a huge increase in the expression of Tet1 and Tet2, as well as Tet3 to a lesser extent (Fig. 6j). Thus, manipulations that promoted the transdifferentiation (p53 knockdown, serum withdrawal and induction of reprogramming factors) synergistically converged on the induction of Tet1 and Tet2. We also compared the impact of basal media and full media on Tet expression by inducing the expression of ANLmp with Dox for 2 days in the two different media. The two media had no significant difference on the mRNA level of Tet1, Tet2 or Tet3 (*P*>0.05, unpaired, two-tailed Student’s *t*-tests; Fig. 6k). This is consistent with the observation that full media promoted neuronal survival rather than conversion *per se* (Fig. 5f).

Using serum withdrawal and full media, MRC5 cells were reprogrammed to iDA neurons with ANLmp (Fig. 6l), ANLmp plus Tet1 (Fig. 6m), Tet2 (Fig. 6n) or Tet3 (Fig. 6o). Tet1, but not Tet2 or Tet3, significantly increased reprogramming yield (Fig. 6p). Tet1 increased the number of DAPI^+^ cells per frame by 47 (from 225 to 272), Tuj1^+^ cells by 50 (from 198 to 248) and TH^+^ cells by 35 (from 130 to 165). Thus, all Tet1-induced increase of DAPI^+^ cells was due to increased number of Tuj1^+^ neurons, 70% of which was TH^+^ neurons. As the loss of cells during reprogramming by ANLmp was visibly reduced by Tet1, the increased numbers of Tuj1^+^ cells, TH^+^ cells and DAPI^+^ cells rendered the ratio of TH^+^/DAPI^+^ or Tuj1^+^/DAPI^+^ not significantly changed (Fig. 6q). The level of 5hmC was significantly increased by induction of ANLmp for 2 days. The effect was further enhanced by Tet1 and abolished by Tet1 shRNA (Fig. 6r). Mouse or human Tet genes had no significant difference in their effects on this transdifferentiation. The expression and localization of exogenous human Tet1, Tet2 and Tet3 (Supplementary Fig. 12) were similar to those of the mouse homologues^47^,^48^. Together, the data suggest that the conversion of human fibroblasts to iDA neurons requires Tet1.

### Functional characterization of iDA neurons

**Figure 7:**
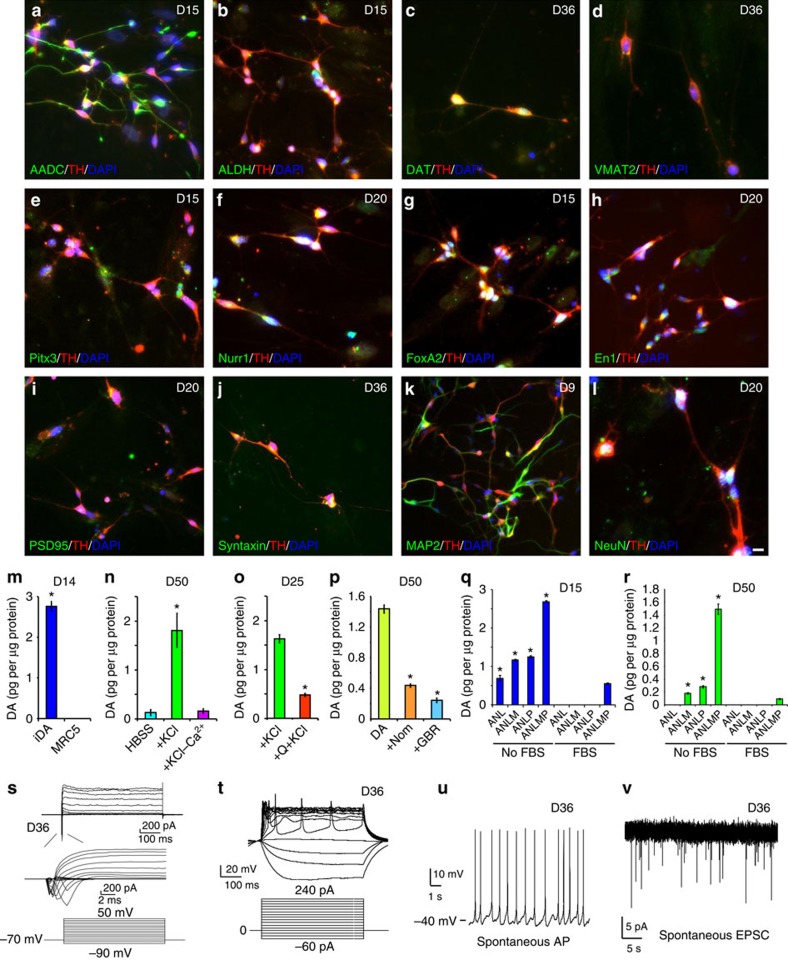
Characterization of iDA neurons. (**a**–**l**) Co-staining of iDA neurons with antibodies against TH and dopaminergic markers AADC (**a**), ALDH1A1 (**b**), DAT (**c**), VMAT2 (**d**), Pitx3 (**e**) and Nurr1 (**f**), or midbrain neuronal markers FoxA2 (**g**) and engrailed 1 (En1) (**h**), or synaptic markers PSD95 (**i**) and syntaxin 1 (**j**), or markers for mature neurons MAP2 (**k**) and NeuN (**l**). Scale bar, 10 μm. (**m**) The amount of endogenous dopamine in the iDA neurons and the original fibroblasts. *P<0.01, unpaired, two-tailed Student’s *t*-tests versus fibroblast; *n*=6 wells from 3 independent experiments. (**n**) Dopamine release from iDA neurons in Hank’s balanced salt solution (HBSS), HBSS with KCl (56 mM) or Ca^2+^-free HBSS with KCl (56 mM). *P<0.01, unpaired, two-tailed Student’s *t*-tests versus HBSS without KCl; *n*=6 wells from 3 independent experiments. (**o**) KCl-induced dopamine release in the absence or presence of the dopamine D2-class agonist quinpirole (Q, 1 μM). *P<0.01, unpaired, two-tailed Student’s *t*-tests; *n*=6. (**p**) Dopamine uptake by iDA neurons for 5 min in the absence or presence of the selective DAT inhibitor nomifensine (10 μM) or GBR12909 (10 μM). *P<0.01, unpaired, two-tailed Student’s *t*-tests versus DA alone; *n*=6 wells from 3 independent experiments. (**q**, **r**) Dopamine content (**q**) or KCl-induced dopamine release (**r**) in iDA neurons generated with various factors and with or without serum withdrawal. FBS, fetal bovine serum. *P<0.01, unpaired, two-tailed Student’s *t*-tests versus corresponding condition with FBS; *n*=6 wells from 3 independent experiments. (**s**) Voltage-gated Na^+^ and K^+^ currents. (**t**) Response to current injections. (**u**) Spontaneous action potentials. (**v**) Spontaneous excitatory postsynaptic currents. Days indicated were from the start of serum withdrawal.

The TH^+^ cells co-expressed dopaminergic markers AADC (Fig. 7a), ALDH1A1 (Fig. 7b), DAT (Fig. 7c), VMAT2 (Fig. 7d), Pitx3 (Fig. 7e), Nurr1 (Fig. 7f), midbrain markers FoxA2 (Fig. 7g) and En1 (Fig. 7h), synaptic markers PSD95 (Fig. 7i) and Syntaxin 1 (Fig. 7j), and mature neuronal markers MAP2 (Fig. 7k) and NeuN (Fig. 7l). Individual channels of the merged images are shown in Supplementary Fig. 13. Co-staining of MRC5-derived iDA neurons at day 10 for Tuj1 and fibroblast markers such as P4HA1 (prolyl 4-hydroxylase, a key enzyme in collagen synthesis; Supplementary Fig. 14b) or vimentin (Supplementary Fig. 14d) showed that there were very few cells faintly expressing P4HA1 and virtually no cells expressing vimentin. In contrast, MRC5 cells at day 0 showed no expression of Tuj1 and very strong expression of P4HA1 (Supplementary Fig. 14a) and vimentin (Supplementary Fig. 14c). We also co-stained iDA neurons at day 10 for Tuj1, MAP2 and DAPI and found that reprogramming efficiency for neurons, as measured by the ratio of either Tuj1/DAPI or MAP2/DAPI, was not significantly different (*P*>0.05, unpaired, two-tailed Student’s *t*-tests; Supplementary Fig. 14e–i). Consistently, when iDA neurons were co-stained for AADC, TH and DAPI, we found that reprogramming efficiency for dopaminergic neurons, as measured by the ratio of either AADC/DAPI or TH/DAPI, was not significantly different (*P*>0.05, unpaired, two-tailed Student’s *t*-tests; Supplementary Fig. 14j–n). These results confirmed the specificity of the Tuj1 and TH antibodies, as well as the validity of our quantification method. The induction of many other neuronal and dopaminergic genes was shown in Supplementary Fig. 15. The induction of microtubule genes (*TUBB3* and *MAP2*) was particularly fast and robust (Supplementary Fig. 15), consistent with the rapid morphological changes (Supplementary Fig. 7d–i). These results suggest that the TH^+^ cells are midbrain dopaminergic neurons.

Dopamine can be readily measured in the iDA neurons, but not in the parental fibroblasts, by high-performance liquid chromatography (HPLC) coupled with electrochemical detection (Fig. 7m). The iDA neurons released dopamine in response to KCl (56 mM for 15 min). The release was completely blocked in Ca^2+^-free Hank’s balanced salt solution (HBSS; Fig. 7n). Dopamine release evoked by KCl (56 mM for 15 min) was significantly decreased by the dopamine D2-class agonist quinpirole (1 μM, 15 min before and 15 min overlapping KCl treatment; Fig. 7o). It demonstrates that the iDA neurons had functional dopamine autoreceptors that serve to downregulate dopamine release^49^. When iDA neurons were treated with quinpirole (1 μM for 10 min), ERK phosphorylation was prominently observed in Tuj1^+^ neurons and TH^+^ neurons. The effect was completely blocked by the D2-class antagonist sulpiride (1 μm for 15 min, of which 5 min was before the addition of quinpirole; Supplementary Fig. 16). The iDA neurons exhibited robust dopamine uptake, which was significantly blocked by the selective inhibitor of dopamine transporter, nomifensine (10 μM) or GBR12909 (10 μM; Fig. 7p).

To demonstrate that enhanced reprogramming efficiency produced a commensurate increase in function, we measured DA content (Fig. 7q) and KCl-evoked DA release (Fig. 7r) and found that they were markedly higher in iDA neurons derived with our new method, compared with the old method with ANL in the absence of G1 arrest^10^ (Fig. 7q,r). Consistently, protein levels of TH, β3-tubulin (Tuj1) and ALDH1A1 were markedly increased by our new method (Supplementary Fig. 17). Electrophysiological recordings showed that the iDA neurons had voltage-dependent Na^+^ and K^+^ currents (Fig. 7s), evoked action potentials (Fig. 7t), spontaneous action potentials (Fig. 7u) and spontaneous excitatory postsynaptic currents (Fig. 7v). As the iDA neurons maturated, the above electrophysiological properties became more robust (Supplementary Fig. 18 and Supplementary Table 1). Together, these results showed that the human fibroblasts were converted to midbrain DA neurons with active synaptic transmission.

## Discussion

This study discovered three kinetic barriers in the direct conversion of human fibroblasts to iDA neurons by Ascl1, Nurr1, Lmx1a (ref. 10) and miR124 (refs 23, 24). First, p53 appears to serve as a major gatekeeper in maintaining the existing transcription network of the cell. When p53 level is reduced by shRNA or MDM2, the conversion became significantly more efficient (Fig. 1). Second, reprogramming of fibroblasts to neurons requires cell cycle exit, as no significant numbers of induced neurons were derived from EdU-labelled cells (Fig. 3). Thus, G1 arrest induced by three independent methods—serum withdrawal (Fig. 3), the CDK2 inhibitor SU9516 (Fig. 4) or the mTOR inhibitor Torin1 (Fig. 4)—all significantly enhanced reprogramming (*P*<0.05, unpaired, two-tailed Student’s *t*-tests). Third, the rapid pace of conversion means that cells need to be cultured in a different environment that can sustain the survival and maturation of neuron-like cells. The first 2 days of reprogramming, during which an intrinsic programme appeared to be executed, did not require full media (Fig. 5f and Supplementary Fig. 8p–r). As the cells acquired neuronal characteristics from day 3 or so, they needed full media for survival and maturation.

Our studies showed that Tet1 played a critical role in the epigenetic conversion of human fibroblasts to iDA neurons. The conversion was obliterated by Tet1 knockdown (Fig. 6). Conditions that enhanced the conversion (p53 knockdown, serum withdrawal or expression of reprogramming factors) significantly induced Tet1 in a synergistic manner (*P*<0.05, unpaired, two-tailed Student’s *t*-tests; Fig. 6). Consistent with these, Tet1 overexpression increased the yield of TH^+^, Tuj1^+^ and DAPI^+^ cells, as cell death was visibly reduced (Fig. 6). It seems likely that Tet1, by pushing the epigenome towards a neuronal state, which has the highest 5hmC content among all cells^20^, may greatly reduce conflicting signals impinging on the cells and thus promote reprogramming and survival of the converted cells. We suspect that the three parameters—cell cycle, p53 and extracellular environment—may be of general importance to cellular reprogramming by defined factors. The systematic identification of the requisite transcription factors, in conjunction with fine tuning of the three parameters, should enable the generation of many types of human cells, and thus tissues, from accessible materials such as fibroblasts.

## Methods

### Materials

Nutlin-3a was purchased from Sigma. Dorsomorphin dihydrochloride, SU9516 and Torin1 were from Tocris. Purmorphamine, CHIR 99021, SB431542 and Y27632 were purchased from Reagents Direct. Recombinant human bFGF, NGF, GDNF, BDNF and TGFβ3 were purchased from PeproTech. FUW-tetO-LoxP, pLKO.1/p53shRNA, pLKO.1/scrambled shRNA, pCAG/p21, pMD2.G and psPAX2 were purchased from Addgene. Human Ascl1 (Genebank accession BC031299), Nurr1 (CV028069) and Lmx1a (BC06635) were purchased from OpenBiosystems and subcloned by PCR to the EcoRI site on the FUW-tetO-LoxP vector. Human miR-124 (MIMAT0000422) was amplified from normal human fibroblast genomic DNA and subcloned to the EcoRI site on the FUW-tetO-LoxP vector. FUW-LoxP-M2rtTA was generated by subcloning the BspEI fragment containing the loxP site from FUW-tetO-Loxp-hOCT4 (Addgene) to the BspEI site on the 3′ long terminal repeat of FUW-M2rtTA (Addgene). Human MDM2 complementary DNA (cDNA) was kindly provided by Xinjiang Wang at Roswell Park Cancer Institute and was subcloned to the EcoRI site on the FUW-tetO-LoxP vector. Human p21 was subcloned to the EcoRI site on the FUW vector. Flag-tagged full-length mouse Tet1, Tet2 or Tet3 cDNA^47^ was cloned in the Dox-inducible lentiviral vector pTYF-TRE. Human Tet1 cDNA was cloned from MRC5-derived iDA neurons. Human Tet2 cDNA was cloned from leukocytes. Human Tet3 cDNA was purchased from Addgene. These cDNAs were subcloned to the FUW-tetO-LoxP lentiviral vector. ShRNA against human Tet1, Tet2 or Tet3 was cloned in pLKO.1. The target sequences for human Tet1, Tet2 and Tet3 are 5′-CCTTGATAGAATCACTCAGTT-3′ (ref. 50), 5′-GGATCATTCTTTGGCCAGA-3′ (ref. 51) and 5′-GGAGAAAGATGAAGGTCCA-3′ (ref. 51), respectively. All constructs were verified by sequencing.

### Generation of iDA neurons from human fibroblasts

Primary human fibroblasts MRC5 and CCD-19 LU (both from American Type Culture Collection), IMR90, AG22056, AG16146 and GM00731 (all from Coriell) were cultured in DMEM containing 10% FBS and 2 mM L-glutamine. All cell cultures were performed without antibiotics and regularly tested for the absence of mycoplasma by PCR. Lentivirus production and fibroblast infection were performed as described previously^25^. Briefly, lentiviruses were produced by cotransfecting 293FT cells (Life Technologies) in 10-cm dishes with 10 μg FUW-tetO-LoxP-cDNA (hAscl1, hNurr1, hLmx1a and hmiR-124), or 10 μg pLKO.1/p53shRNA, or 10 μg FUW-LoxP-M2rtTA with 2.5 μg pMD2.G and 7.5 μg psPAX2 using Lipofectamine 2000. Lentiviruses generated with the pTYF vector were produced by cotransfection of pTYF-mTet1, mTet2 or mTet3 with pNHP, pHEF-VSVG and pCEP4-Tat in 293FT cells. Viruses were collected from 16 to 60 h after transfection and titred for p24 levels using an ELISA kit (ZeptoMetrix Corporation, Buffalo, NY). After the fibroblasts were thawed and passaged once more, they were plated at 5 × 10^3^ cm^−2^ and infected 1 day later for 16 h with the indicated combinations of lentiviruses (hASCL1, hNurr1, hLmx1a, hmiR-124 or hp53shRNA each at MOI 10, M2rtTA at MOI 20) in the presence of 8 μg ml^−1^ polybrene. Virus-containing media was removed after 16 h and replaced with DMEM containing 10% FBS (which was omitted in experiments with cell cycle arrest), 2 mM L-glutamine and doxycycline (1 μg ml^−1^). After 24 h, the media was changed to neural induction media (DMEM/F12, 1 × N2 supplements, 1 × NEAA, 1 × B27, 20 ng ml^−1^ bFGF or NGF, 1 μM dorsomorphin, 10 μM SB431542, 3 μM CHIR99021, 2 μM purmorphamine, 0.2 mM vitamin C, 10 μM ROCK inhibitor Y27632, 20 ng ml^−1^ GDNF, 20 ng ml^−1^ BDNF and 1 ng ml^−1^ TGFβ3) containing 1 μg ml^−1^ doxycycline. The media was changed every other day for the duration of the culture period. After 6 days’ induction, dorsomorphin, SB431542, CHIR99021, purmorphamine and Dox were removed. In some experiments, we used full media, which is neural induction media minus bFGF and plus NGF (20 ng ml^−1^). Most experiments were completed at day 10. For experiments that went beyond 10 days, all media additives except GDNF, BDNF and TGFβ3 were removed from the full media from day 11 onward. In some experiments, basal media (DMEM/F12, 1 × N2 supplements, 1 × NEAA and 1 × B27) was used instead.

### Cell cycle analysis

Cell cycle profiles were analysed by the standard propidium iodide staining protocol. Briefly, cells were collected by trypsinization at indicated time points, fixed with cold 70% ethanol at −20 °C for at least 2 h and stained in propidium iodide master mix (40 μg ml^−1^ propidium iodide, 100 μg ml^−1^ DNase-free RNase and 0.1% (v/v) Triton X-100) at 37 °C for 30 min before analysis by flow cytometry. A minimum of 10,000 cells in each sample was sorted on a Becton-Dickinson FACS Caliber flow cytometer (San Jose, CA). Cell cycle was analysed using the CellQuest software (Becton-Dickinson) and plotted using FCS Express 4 software.

### EdU incorporation assay

EdU labelling was performed using a kit from Invitrogen. Briefly, fibroblasts were incubated in media containing 10 μM EdU at the specified time (0, 24 or 48 h after Dox induction) for 2 h. EdU pulse labelling for 2 or 24 h achieved the same effect, as the compound cannot diffuse out of the cells readily once it is taken up through selective transporters. After the cells were washed with regular media three times, they were cultured for another 24 h or 9 days before they were fixed in 4% paraformaldehyde, permeabilized with 0.1% Triton X-100 for 20 min at room temperature and stained in the detection cocktail (4 mM copper sulfate, 40 mM sodium ascorbate and 20 μM Alexa Fluor 488 azide, 20 mM Tris, pH=7.6). Cells were counterstained with DAPI to identify nuclei.

### Array comparative genomic hybridization analysis

Genomic DNA was extracted from MRC5 fibroblasts or iDA neurons at day 12 using QIAamp DNA Mini Kit (Qiagen). Array comparative genomic hybridization of the two samples was performed by the Genomics Shared Resource at Roswell Park Cancer Institute using Agilent Human Genome CGH Microarray 244A and scanned on Agilent Technologies Scanner G2505C. There are 236,381 oligonucleotide probes that are 60 nucleotides in length. The overall median probe spacing is 8.9 kb (7.4 kb median spacing in Refseq genes).

### Dopamine release

DA release was measured as previously described^25^. Briefly, iDA neurons cultured in six-well plates were incubated at 37 °C in 1 ml HBSS for 30 min or in 1 ml HBSS for 15 min and then 56 mM KCl was added for another 15 min, or in 1 ml HBSS without Ca^2+^ and without Mg^2+^, but with 2 mM EDTA for 15 min and then 56 mM KCl was added for another 15 min. The 1-ml HBSS solutions were taken out from the wells. The amounts of DA in HBSS solutions were measured by reverse phase HPLC (ESA Model 582 with ESA MD150 × 3.2 column, at 0.6 ml min^−1^ flow rate in MD-TM mobile phase) coupled with electrochemical detection (ESA Coulochem III, E1: −150 mV, 2 μA; E2: 220 mV, 2 μA). Cells in the wells were lysed in 0.5 N NaOH to measure protein levels, which were used to normalize dopamine release.

### Dopamine uptake

DA uptake was measured as previously described^25^. Briefly, iDA neurons cultured in six-well plates were rinsed with 1 ml prewarmed uptake buffer (10 mM HEPES, 130 mM NaCl, 1.3 mM KCl, 2.2 mM CaCl_2_, 1.2 mM MgSO_4_, 1.2 mM KH_2_PO_4_, 10 mM glucose, pH 7.4) three times. Cells were incubated for 5 min at 37 °C with 1 ml uptake buffer containing 5 μM dopamine without or with 10 μM nomifensine or 10 μM GBR12909 (both are selective inhibitors of dopamine transporter). After the cells were washed at least three times in uptake buffer, they were lysed in 0.1 M perchloric acid with 1 mM EDTA and 0.1 mM sodium bisulfite. Cleared cell lysates were analysed for dopamine on HPLC coupled with electrochemical detection (E1: −150 mV, 2 μA; E2: 220 mV, 2 μA). The pellets of cellular proteins were dissolved in 1 ml 0.5 N NaOH to measure protein contents, which were used to normalize dopamine uptake. The amount of endogenous dopamine in iDA neurons without any treatment was also measured.

### Immunocytochemistry and morphological analysis of neurites

Cells grown in 12-well plates were fixed *in situ* with 4% paraformaldehyde in PBS for 20 min, permeabilized with 0.1% Triton X-100 in PBS for 15 min at room temperature, blocked in 3% BSA in PBS for 60 min at room temperature and then incubated in primary antibody overnight at 4 °C, secondary antibody for 2 h at room temperature. The sources, catalogue numbers and dilutions of the antibodies used in this study are listed in Supplementary Table 2. Fluorescence images were taken on Zeiss Axio Observer Inverted Microscope with lenses corrected for plastic culture plates. TH^+^, Tuj1^+^ and DAPI^+^ cells were counted blindly by a different person from 10 randomly selected images at × 10 magnification for each condition. In all, 30–60 TH^+^ neurons from three independent experiments for each condition were traced and analysed blindly for total neurite length using NIH ImageJ with the NeuronJ plugin^26^. The number of neurites was counted manually after tracing. To avoid any ambiguity, neurons with overlapping neurites were excluded from analysis.

### Real time quantitative RT–PCR

Total RNA was extracted using RNeasy Mini kit (Qiagen). Further DNA removal was performed with the RNase-Free DNase Set (Qiagen). First-strand cDNA was synthesized with oligo dT or random hexamers as primers, using SuperScript First-Strand Synthesis System according to the manufacturer’s protocol (Life Technologies). An equal volume mixture of the products was used as templates for PCR amplification. Reactions were performed in a 25-μl volume with iQ SYBR Green Supermix (Bio-Rad) and 200 nM each of forward and reverse primers shown in Supplementary Table 3 using iCyler and iQ software (Bio-Rad). Each sample was run in triplicate. PCR conditions included an initial denaturation step of 4 min at 95°C, followed by 40 cycles of PCR consisting of 30 s at 95 °C, 30 s at 60 °C and 30 s at 72 °C. Average threshold cycle (Ct) values from the triplicate PCR reactions for a gene of interest were normalized against the average Ct values for GAPDH from the same cDNA sample.

### 5hmC dot blot

Genomic DNA was extracted with QIAamp DNA mini kit (Qiagen). A unit of 200 or 500 ng genomic DNA was denatured in 0.1 M NaOH at 100 °C for 10 min and followed by the addition of an equal volume of cold 2 M ammonium acetate (pH 7.2). Denatured DNA samples were spotted on a nitrocellulose membrane and crosslinked by ultraviolet with Stratalinker 2400 twice. The membrane was blocked with 5% non-fat milk for 1 h and incubated with anti-5hmC for detection by ECL.

### Electrophysiology

Recordings of spontaneous EPSC (sEPSC) used standard whole-cell voltage-clamp techniques^25^,^52^. The membrane potential was held at −70 mV. The external solution contained (in mM): 130 NaCl, 26 NaHCO_3_, 3 KCl, 5 MgCl_2_, 1 CaCl_2_, 1.25 NaH_2_PO_4_ and 10 glucose, pH 7.3–7.4, 300–305 mOsm. To isolate AMPAR-mediated response, the NMDA receptor antagonist D-aminophosphonovalerate (50 μM) and GABA_A_ receptor antagonist bicuculline (10 μM) were added. The internal solution consisted of the following (in mM): 130 Cs methanesulfonate, 10 CsCl, 4 NaCl, 10 HEPES, 1 MgCl_2_, 5 EGTA, 2.2 QX-314, 12 phosphocreatine, 5 MgATP, 0.2 Na_2_GTP and 0.1 leupeptin, pH 7.2–7.3, 265–270 mOsm. For the recording of action potentials, whole-cell current-clamp recordings were performed with the internal solution containing (in mM): 125 K-gluconate, 10 KCl, 10 HEPES, 0.5 EGTA, 3 Na_2_ATP, 0.5 Na_2_GTP and 12 phosphocreatine, pH 7.25, 280 mOsm. Cells were perfused with ACSF, and membrane potentials were kept at −55 to −65 mV. A series of hyperpolarizing and depolarizing step currents were injected to measure intrinsic properties and to elicit action potentials. Spontaneous action potentials were recorded without current injection. For the recording of voltage-dependent sodium and potassium currents, cells (held at −70 mV) were perfused with artificial cerebrospinal fluid (ACSF), and voltage steps ranging from −90 to +50 mV were delivered at 10-mV increments. Data analyses were performed with Clampfit (Axon instruments) and Kaleidagraph (Albeck Software).

### Statistical analyses

All statistical analyses were done with the software Origin (OriginLab, Northampton, MA). The data were expressed as mean±s.e.m. (standard error of measurement). Unpaired, two-tailed Student’s *t*-tests were performed to evaluate whether two groups were significantly different from each other. Sample size is chosen based on previous studies in the field with similar measurements. No sample is excluded in our analysis.

## Additional information

**Accession codes:** The array comparative genomic hybridization data have been deposited in the GEO database under accession code GSE52352.

**How to cite this article:** Jiang, H. *et al.* Cell cycle and p53 gate the direct conversion of human fibroblasts to dopaminergic neurons. *Nat. Commun.* 6:10100 doi: 10.1038/ncomms10100 (2015).

## Supplementary Material

Supplementary Information Supplementary Figures 1-18 and Supplementary Tables 1-3
