# Author Correction: Cell cycle and p53 gate the direct conversion of human fibroblasts to dopaminergic neurons

**DOI:** 10.1038/s41467-026-77919-w

**Published:** 2026-10-09

**Authors:** Houbo Jiang, Zhimin Xu, Ping Zhong, Yong Ren, Gaoyang Liang, Haley A. Schilling, Zihua Hu, Yi Zhang, Xiaomin Wang, Shengdi Chen, Zhen Yan, Jian Feng

**Affiliations:** 1 Veterans Affairs Western New York Healthcare System, Buffalo, New York 14215 USA; 2 Department of Physiology and Biophysics, State University of New York at Buffalo, Buffalo, New York 14214 USA; 3 Department of Neurology and Institute of Neurology, Ruijin Hospital Affiliated to Shanghai Jiao Tong University School of Medicine, Shanghai, 200025 China; 4 Howard Hughes Medical Institute, Departments of Genetics & Pediatrics, Harvard Medical School, Boston, Massachusetts 02115 USA; 5 Center for Computational Research, New York State Center of Excellence in Bioinformatics & Life Sciences, State University of New York at Buffalo, Buffalo, New York 14260 USA; 6 Department of Neurobiology, Key Laboratory for Neurodegenerative Disorders of the Ministry of Education, Beijing Institute for Brain Disorders, Capital Medical University, Beijing, 100069 China

Correction to: *Nature Communications* https://doi.org/10.1038/ncomms10100, published online 7 December 2015

In the version of this article initially published, the image shown in Fig. 4n incorrectly presented a shifted view of the image shown in Fig. 4m. Due to the age of the article, the figure cannot be updated directly. The corrected Fig. 4n is available as Supplementary Information alongside this amendment. This notice serves to correct the article.

## Supplementary information

Corrected Fig. 4n

