# Supplementary material for: Cell cycle and p53 gate the direct conversion of human fibroblasts to dopaminergic neurons
Source: Nat Commun. 2015 Dec 7;6:10100. doi: 10.1038/ncomms10100 (PMC4672381; doi:10.1038/ncomms10100)
Supplement: Supplementary Information — Supplementary Figures 1-18 and Supplementary Tables 1-3 [file ncomms10100-s1.pdf]

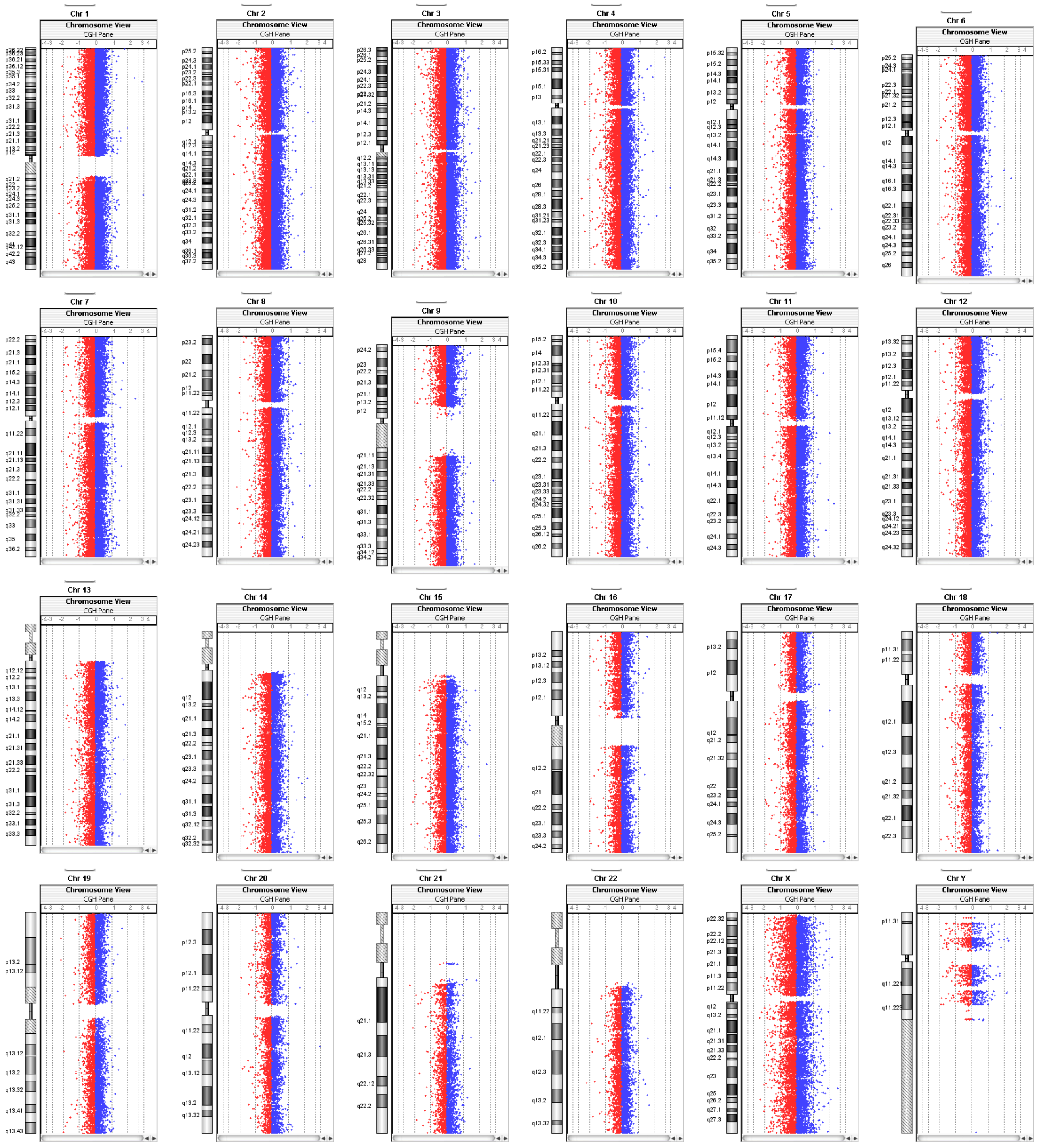

Supplementary Figure 1. aCGH analysis of genomic DNA from MRC5 fibroblasts and iDA neurons. iDA neurons were generated from MRC5 fibroblasts using the best method described in Fig. 5e. Genomic DNA from iDA neurons at day 12 were compared to genomic DNA from the original MRC5 fibroblasts using array Comparative Genomic Hybridization.

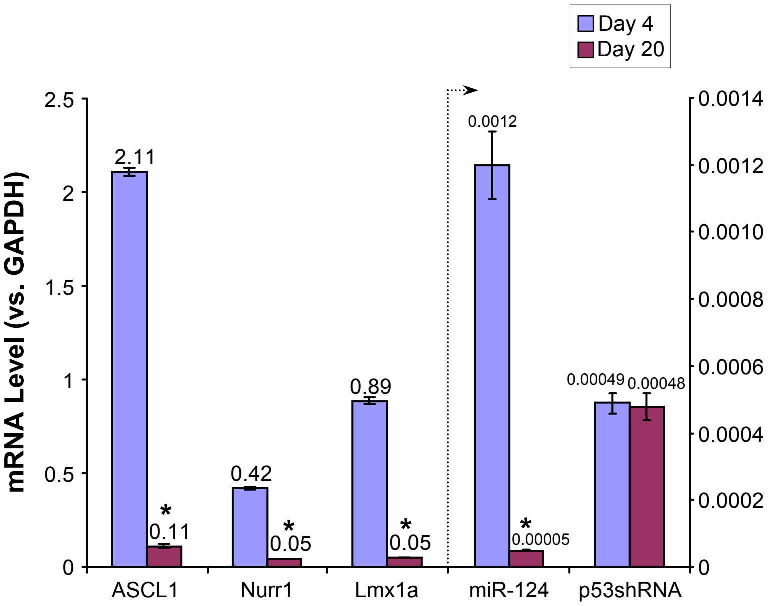

Supplementary Figure 2. Silencing of transgenes. Real-time quantitative RT-PCR measurement of transgene expression levels at day 4 and day 20 of reprogramming. Expression levels are relative to GAPDH levels. The levels of miR124 and p53 shRNA transgenes are according to the right axis. *, $p < 0.01$, unpaired, two tailed Student's t-tests, vs. the preceding bar, n=6 from 3 independent experiments.

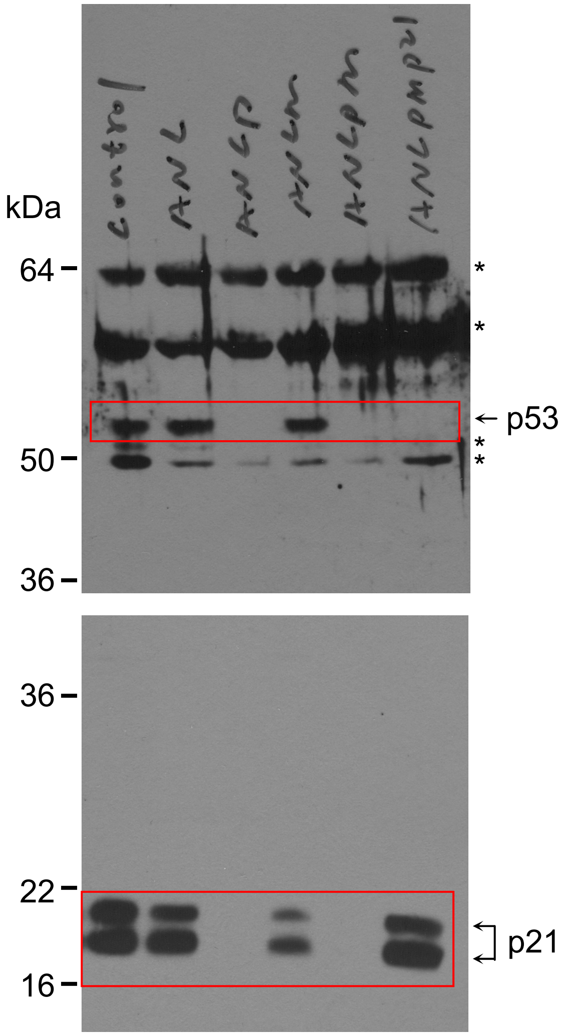

Supplementary Figure 3. Full Western blotting images for p53 and p21. The images correspond to Fig. 2b. Positions of p53 and p21 are indicated by arrows. Red boxes indicate how the images were cropped to produce Fig. 2b. The specificity of the p53 band is corroborated by the size and the effect of p53 knockdown in the 3rd, 5th and 6th lanes. *, non-specific bands. The smearing and dragging effects in the p53 blot were always seen, due to unknown reasons. No such effect was seen for p21 blot on the same set of samples. The sequence of lanes for both p53 and p21 blots were according to the marking on top.

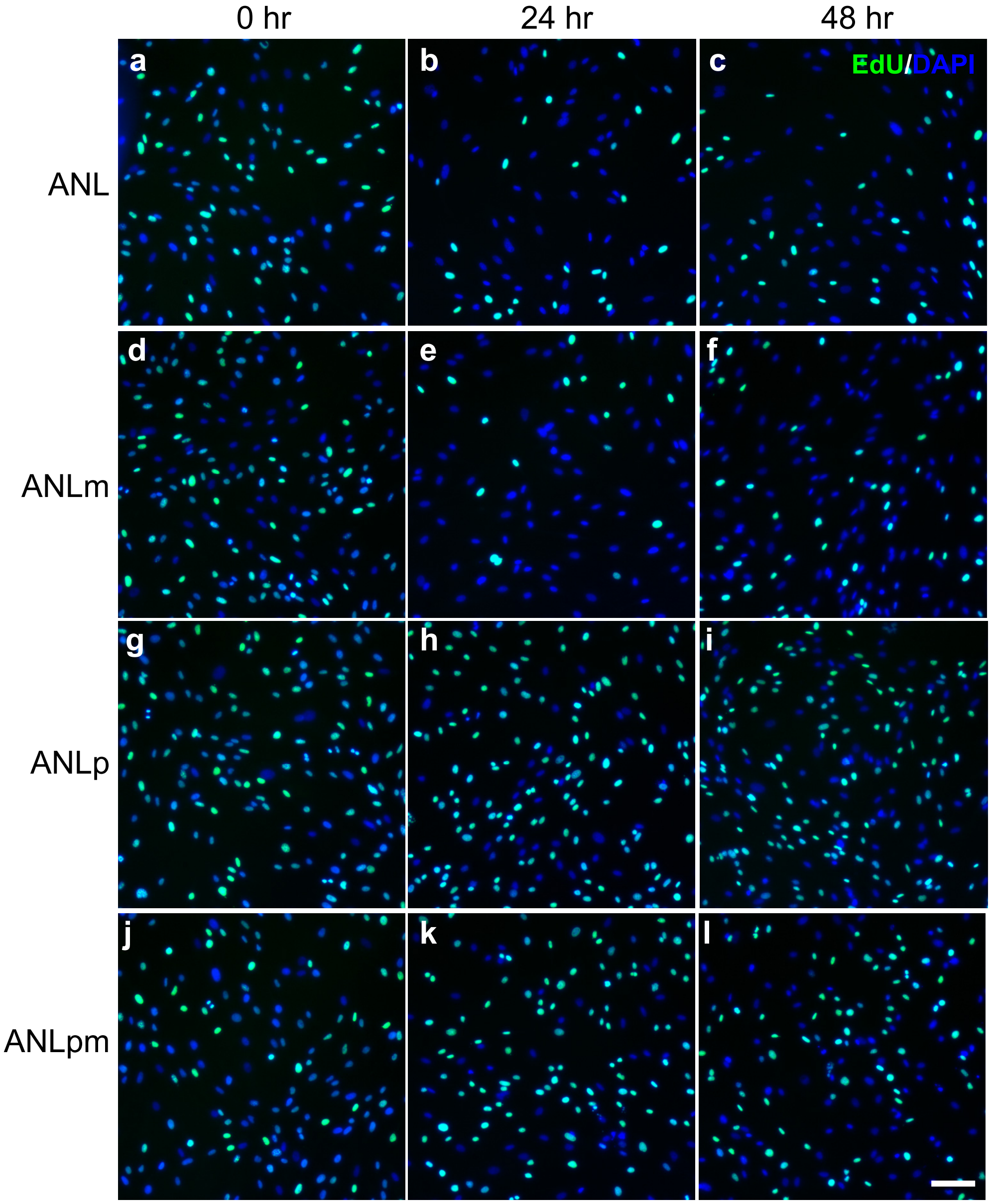

Supplementary Figure 4. EdU labeling in MRC5 cells infected with the indicated viruses at various time points of DOX treatment in Fig. 3a. Cells were pulse-labeled with EdU for 2hr at the indicated time of DOX treatment. After a chase in media without EdU for 24 hr, cells were stained for EdU and DAPI. Bar, 100 μm.

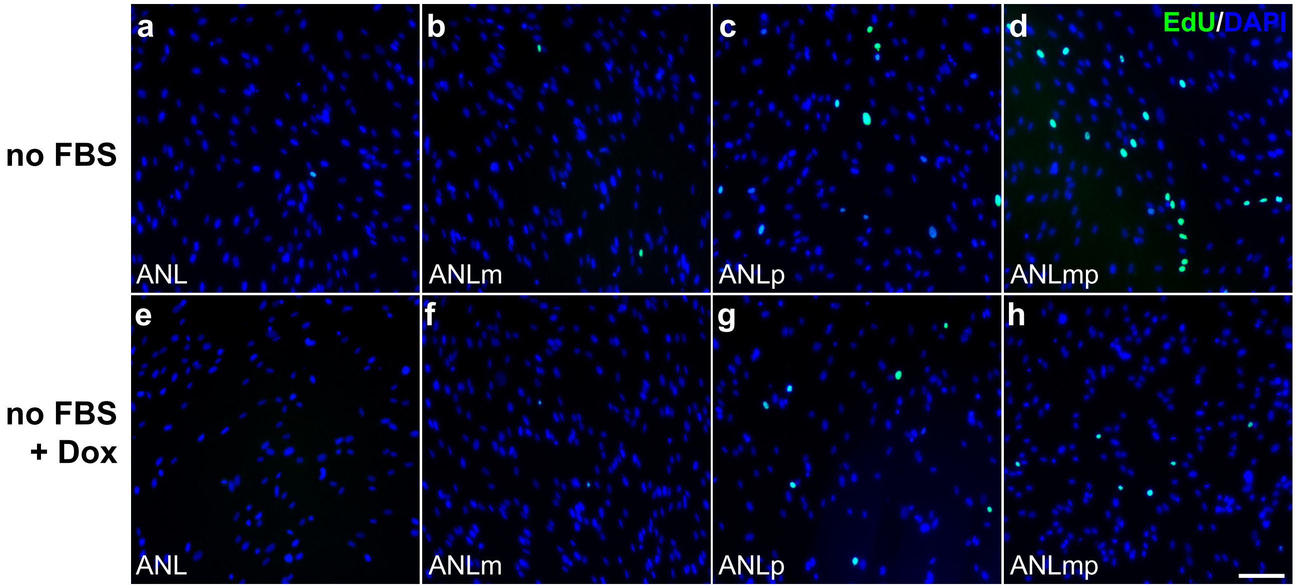

Supplementary Figure 5. EdU labeling with serum withdrawal and DOX treatment. (**a**-**d**) MRC5 cells were infected with the indicated viruses and cultured without serum for 24 hrs. They were pulse-labeled with EdU for 2 hrs and fixed 24 hrs later for EdU and DAPI staining. (**e**-**h**) MRC5 cells were infected with the indicated viruses and cultured without serum for 24 hrs. They were treated with Dox for another 24 hrs then labeled with Edu for 2 hrs. Cells were fixed 24 hrs after the EdU pulse-labeling and stained for EdU and DAPI. Bar, 100 μm.

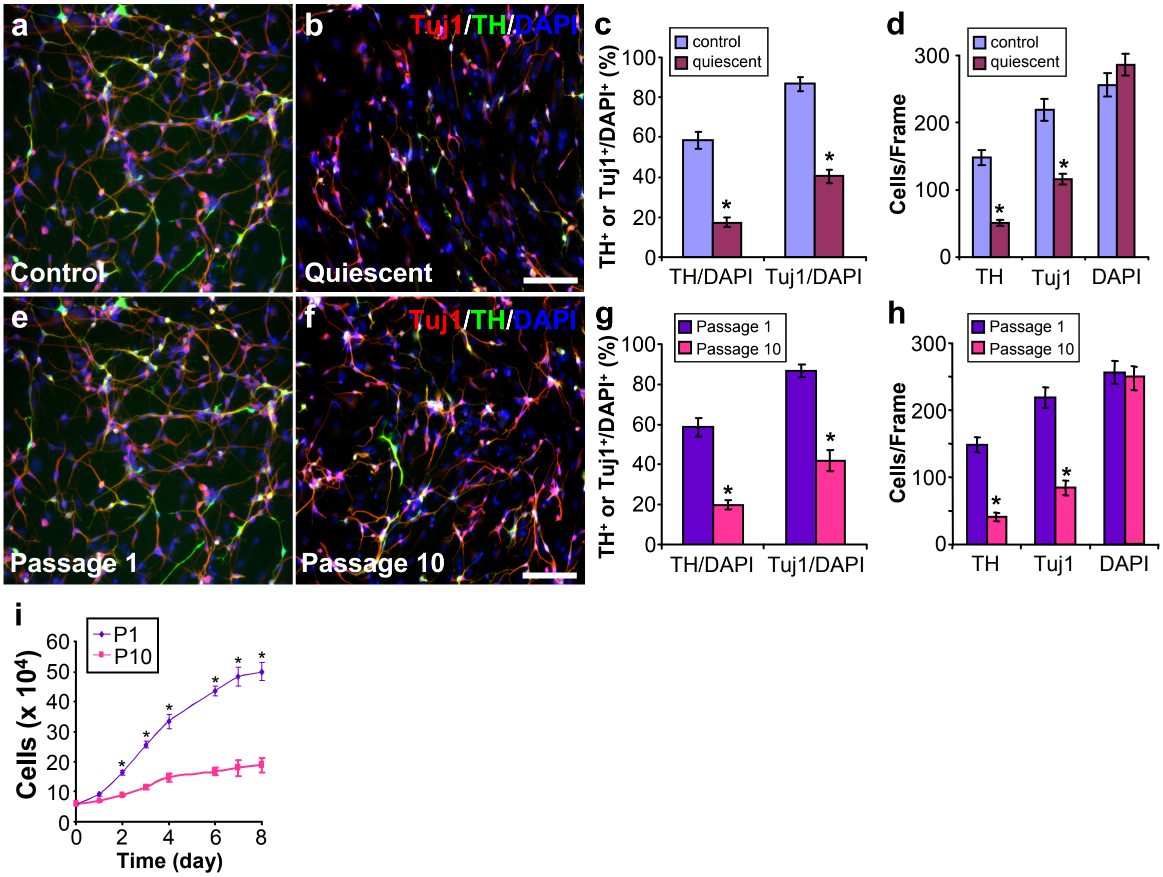

Supplementary Figure 6. Reprogramming of quiescent and senescent fibroblasts. (a-b) Representative images of iDA neurons reprogrammed from control MRC5 cells (**a**) or from MRC5 cells induced to quiescence by culturing in 0.1% FBS for four days (**b**). (**c-d**) The percentages of TH^+ or $Tuj1^+$ neurons in all cells ($DAPI^+$) at day 10 of reprogramming (**c**) and the number of TH^+, $Tuj1^+$, $DAPI^+$ cells per frame of view (**d**) were quantified. *, $p < 0.05$, vs. control, n=6 wells from three independent experiments. Bar, 100 μm. **(e-h)** Representative images of iDA neurons reprogrammed from MRC5 cells at an arbitrary passage 1 (**e**) or from MRC5 cells induced to senescence by additional passaging to passage 10 (**f**). (**g**, **h**) The percentages of TH^+ or $Tuj1^+$ neurons in all cells ($DAPI^+$) at day 10 of reprogramming (**g**) and the number of TH^+, $Tuj1^+$, $DAPI^+$ cells per frame of view (**h**) were quantified. *, $p < 0.05$, vs. passage 1, n=6 wells from three independent experiments. Bar, 100 mm. (**i**) Cell proliferation curves of MRC5 cells at arbitrary passage 1 (P1) or passage 10 (P10). Unpaired, two tailed Student's t-tests were used to calculate all p values.

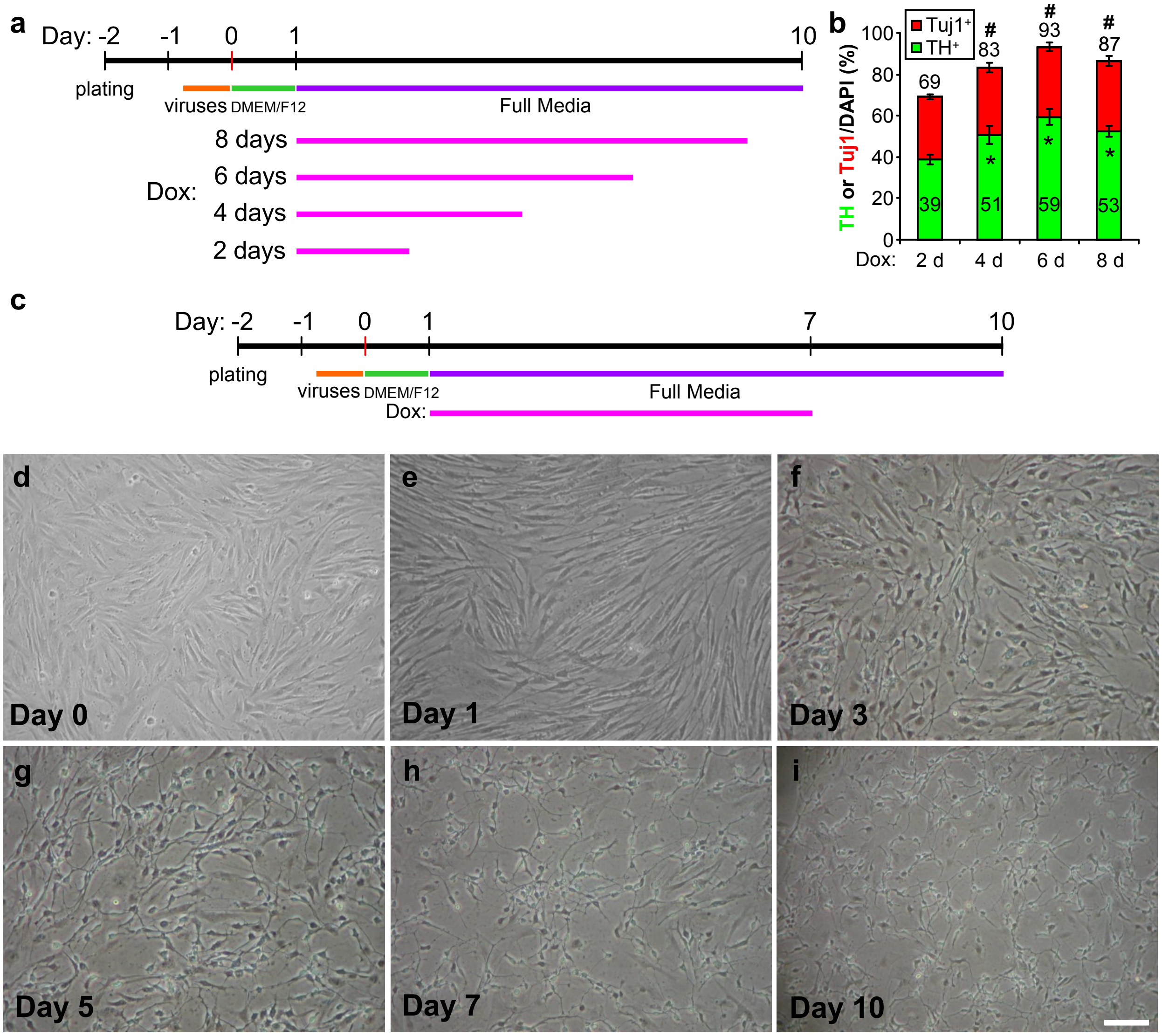

Supplementary Figure 7. Optimal duration of Dox treatment and morphological changes along reprogramming timeline. (**a**) MRC5 cells were infected with ANLmp and synchronized in G1 phase with serum withdrawal for 24 hrs. Doxycycline (Dox, 1μg/ml) were added in Full Media for the indicated durations. (**b**) The percentage of TH^+ or $Tuj1^+$ cells in $DAPI^+$ cells at day 10. *, #, $p < 0.05$, unpaired, two tailed Student's t-tests, vs. day 2 for TH^+ or $Tuj1^+$ neurons, respectively, n=6 wells from three independent experiments. (**c**-**i**) Using the protocol in (**c**), ANLmp-infected MRC5 human fibroblasts (**d**) were synchronized in G1 phase with serum withdrawal (**e**). After Dox treatment in Full Media, phase contrast images were taken at the indicated days (**f-i**). Bar, 100 μm.

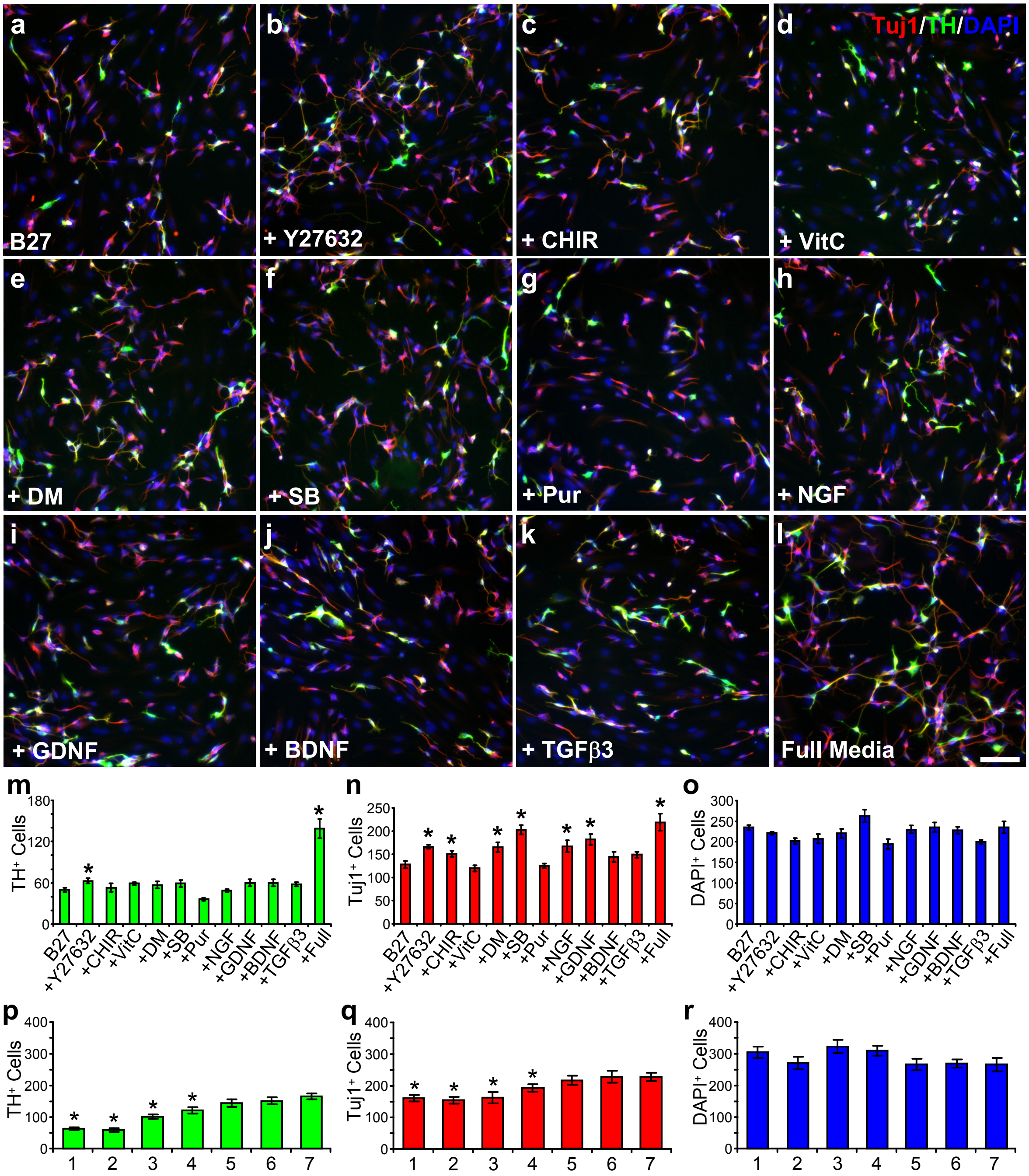

Supplementary Figure 8. Effects of media supplements on the transdifferentiation. (**a-l**) Representative images of converted cells cultured with the indicated media supplements. Bar, 100 μm. (**m-o**) Numbers of TH^+ (**m**), $Tuj1^+$ (**n**) or $DAPI^+$ (**o**) cells per view for the indicated condition in Fig. 5e. *, $p<0.05$, vs. B27, n=6 wells from 3 independent experiments. B27, B27 supplements; VitC, vitamin C; DM, dorsomorphin; SB, SB431542; CHIR, CHIR99021; Pur, Purmorphamine; NGF, nerve growth factor; GDNF, glial cell line-derived neurotrophic factor; BDNF, brain-derived neurotrophic factor; TGFβ3, transforming growth factor β3; Y27632, Rock inhibitor; Full, all of the above; +, B27 plus the indicated supplement. (**p-r**) Numbers of TH^+ (**p**), $Tuj1^+$ (**q**) or $DAPI^+$ (**r**) cells per view for conditions 1 to 7 in Figure 5f. *, $p<0.05$, vs. condition 7, n=6 wells from 3 independent experiments. Unpaired, two tailed Student's t-tests were used to calculate all p values.

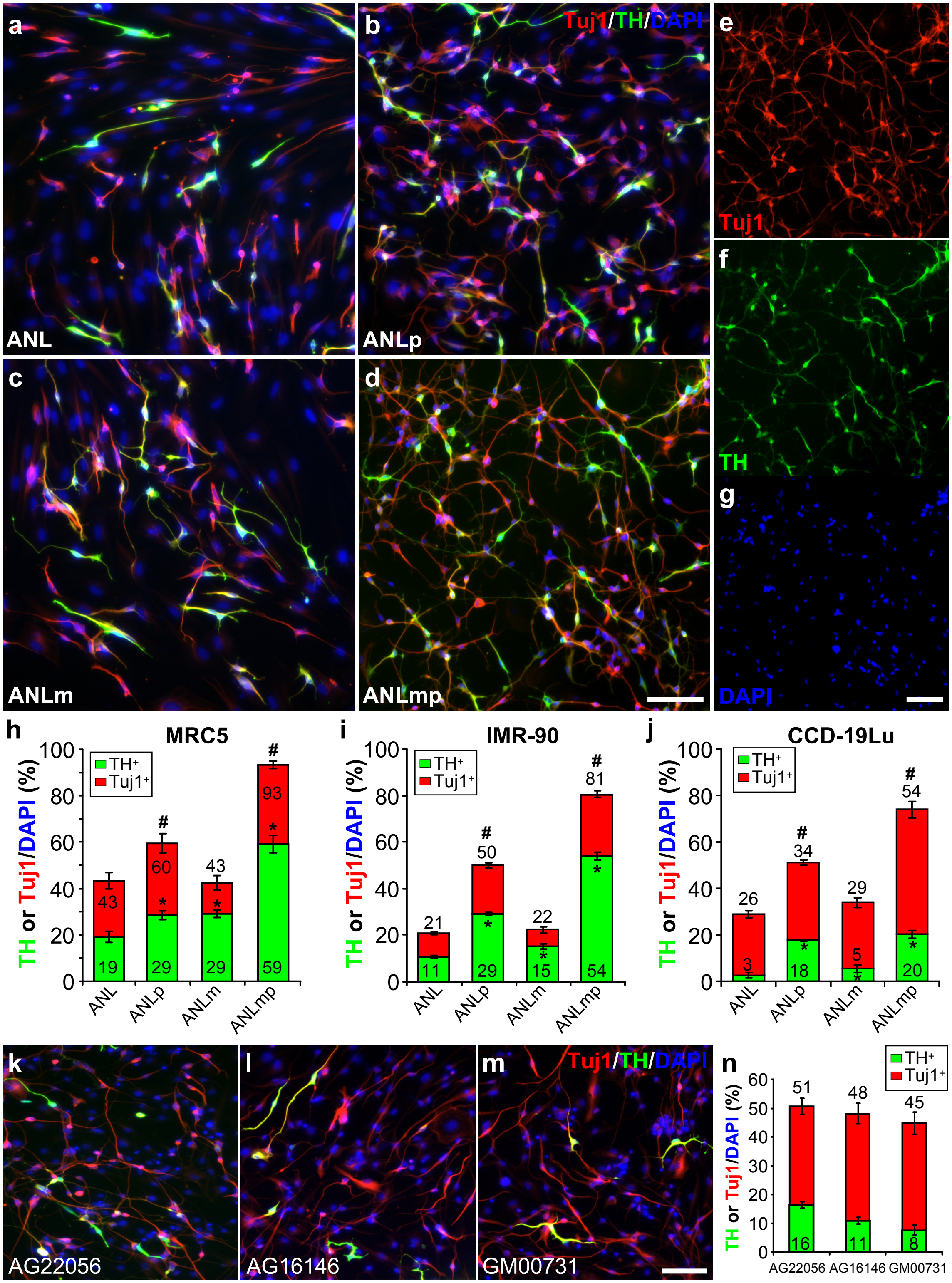

Supplementary Figure 9. Reprogramming efficiencies of different combinations of viruses in various human fibroblasts. (**a-d**), Representative image of iDA neurons generated from MRC5 cells with ANL (**a**), ANLp (**b**), ANLm (**c**), ANLmp (**d**) using serum withdrawal and Full Media. Bar, 100 µm. (**e-g**) Separate channels of (**d**). (**h-j**) Quantification of data for human fetal fibroblasts MRC5 (**h**), human fetal fibroblasts IMR-90 (**i**), and human adult fibroblasts CCD-19Lu (**j**). *, #, $p < 0.05$ vs. ANL for TH^+ or $Tuj1^+$ neurons, respectively, n=6 wells from three independent experiments. (**k-n**) iDA neurons derived from additional fibroblasts from subjects at age 0 (**k**), 31 (**l**) and 96 (**m**) using ANLmp and the quantification of reprogramming efficiency (**n**). Bars, 100 µm. Unpaired, two tailed Student's t-tests were used to calculate all *p* values.

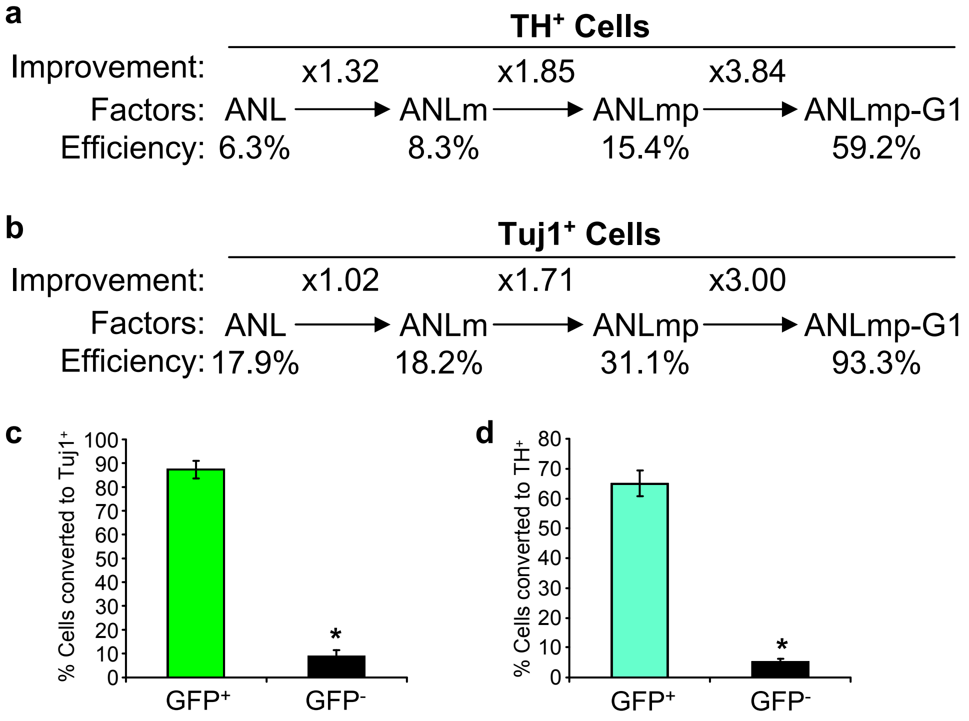

Supplementary Figure 10. Relative contributions of miR124, p53 knockdown and G1 arrest to reprogramming efficiency and ascertaining the effect of p53 knockdown. (a-b) The percentages of TH^+ cells (**a**) or $Tuj1^+$ cells (**b**) in $DAPI^+$ cells were listed for various conditions to calculate the improvement in reprogramming efficiency provided by miR124, p53 knockdown or G1 arrest. **(c-d)** MRC5 cells were reprogrammed with ANLm plus p53shRNA in a bicistronic vector expressing GFP (pLKO.3G) under serum starvation condition and full media. The percentage of $Tuj1^+$ cells in GFP^+ or GFP^- cells (**c**) and the percentage of TH^+ cells in GFP^+ or GFP^- cells (**d**) showed that reprogramming was markedly increased in cells infected with p53 shRNA, compared to that in cells not infected with p53 shRNA. *, $p < 0.001$, vs. GFP^+, n=6 wells from 3 independent experiments. Unpaired, two tailed Student’s t-tests were used to calculate all p values.

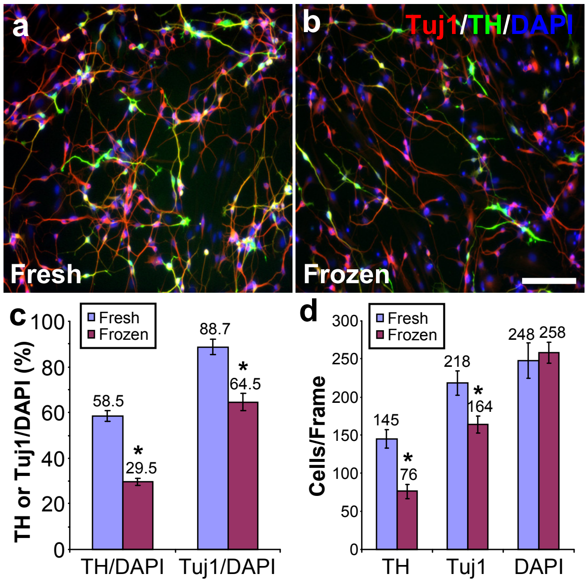

Supplementary Figure 11. Reprogramming of cells frozen after infection. (a, b) MRC5 cells infected with ANLmp lentiviruses were cultured without serum for 24 hours and reprogrammed to iDA neurons either right away (a) or after being frozen in liquid nitrogen for 7 days (b). **(c)** The percentage of TH^+ or $Tuj1^+$ cells in total cells ($DAPI^+$) at day 10 of reprogramming. (**d**) The number of TH^+, $Tuj1^+$ or $DAPI^+$ cells per frame of view at day 10 of reprogramming. *, $p < 0.05$, vs. the preceding bar, n=6 wells from 3 independent experiments. Bar, 100 μm. Unpaired, two tailed Student's t-tests were used to calculate all *p* values.

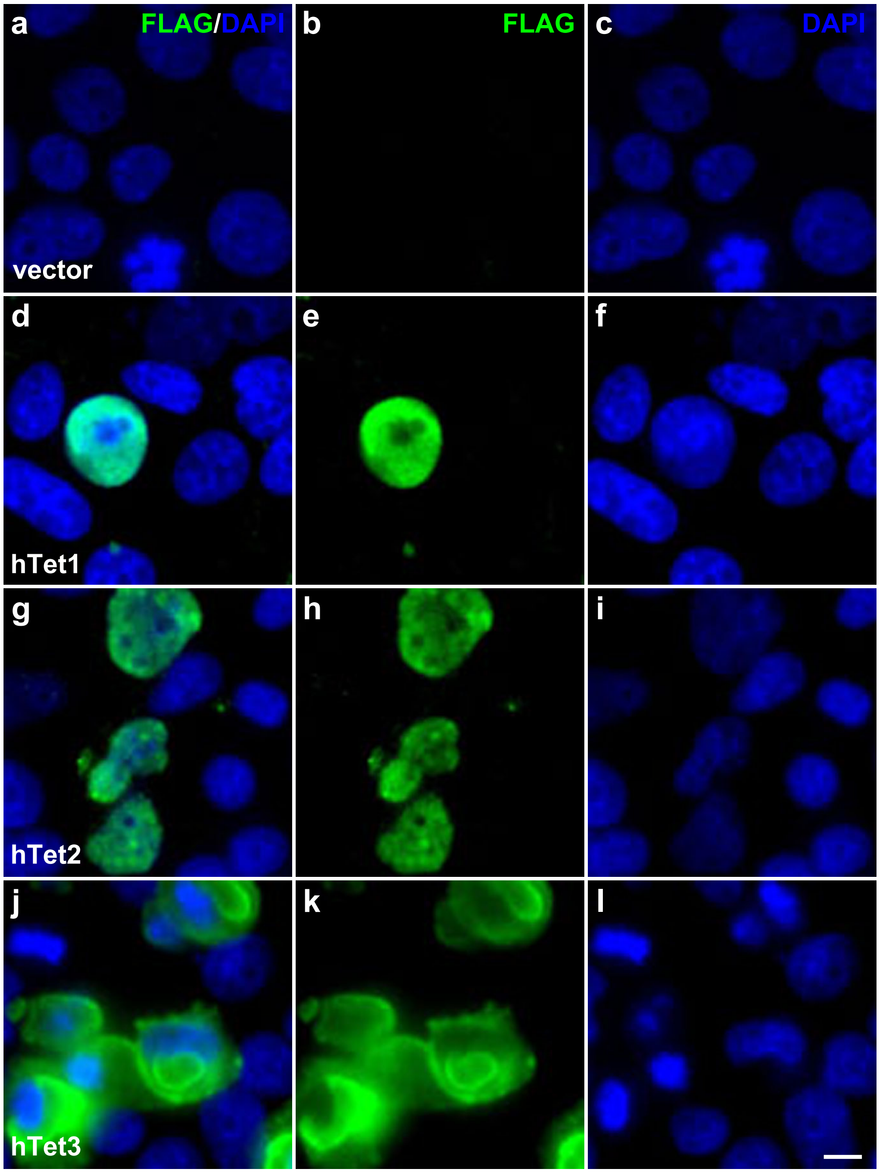

Supplementary Figure 12. The expression and localization of human Tet proteins. **(a-l)** 293FT cells were transfected with empty vector or mammalian expression construct for FLAG-tagged human Tet1, Tet2 or Tet3. Cells were costained for FLAG and DAPI. Bar, 10μm.

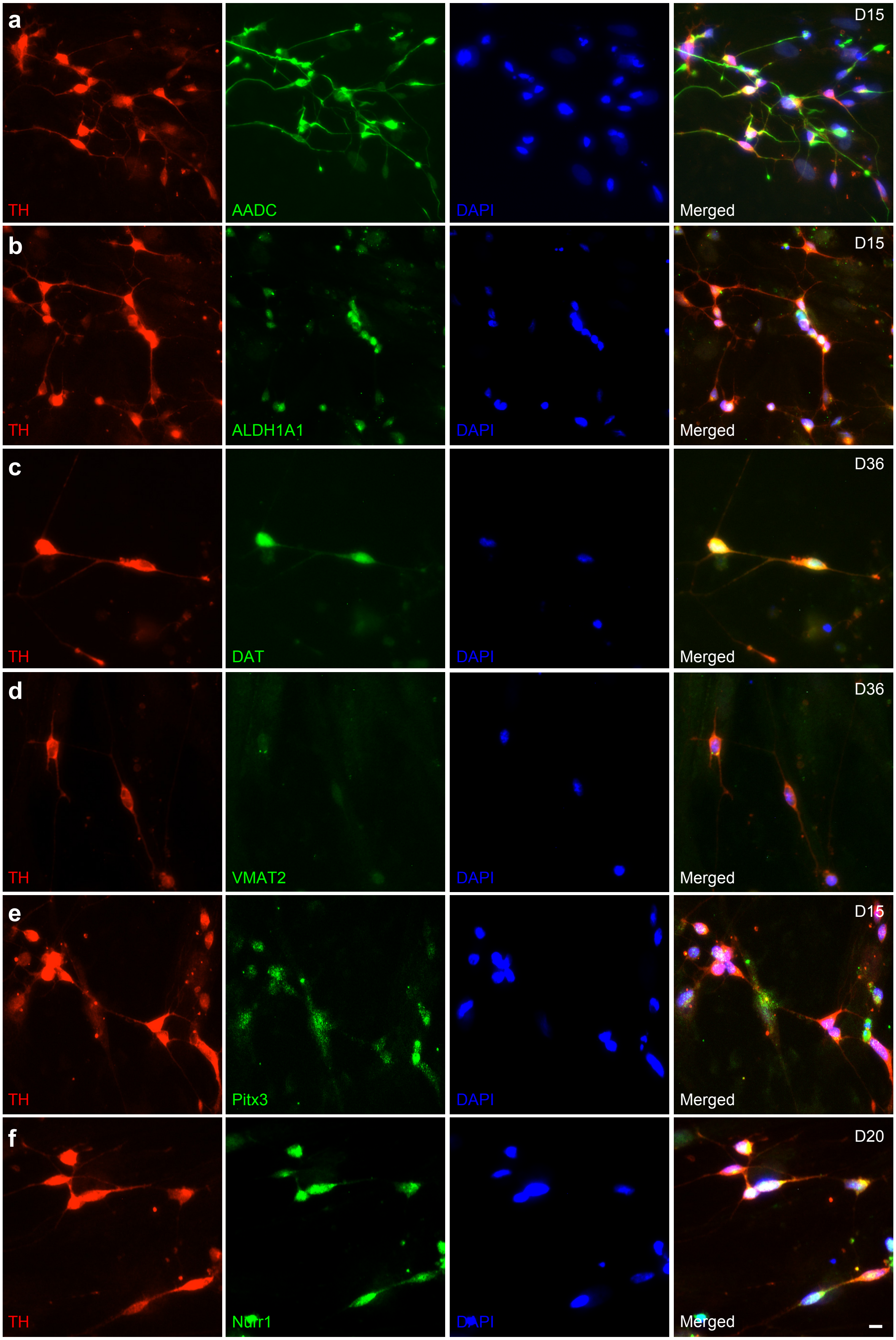

Supplementary Figure 13a-f. Separate channels of images in Fig. 7a-f. Bar, 10 μm.

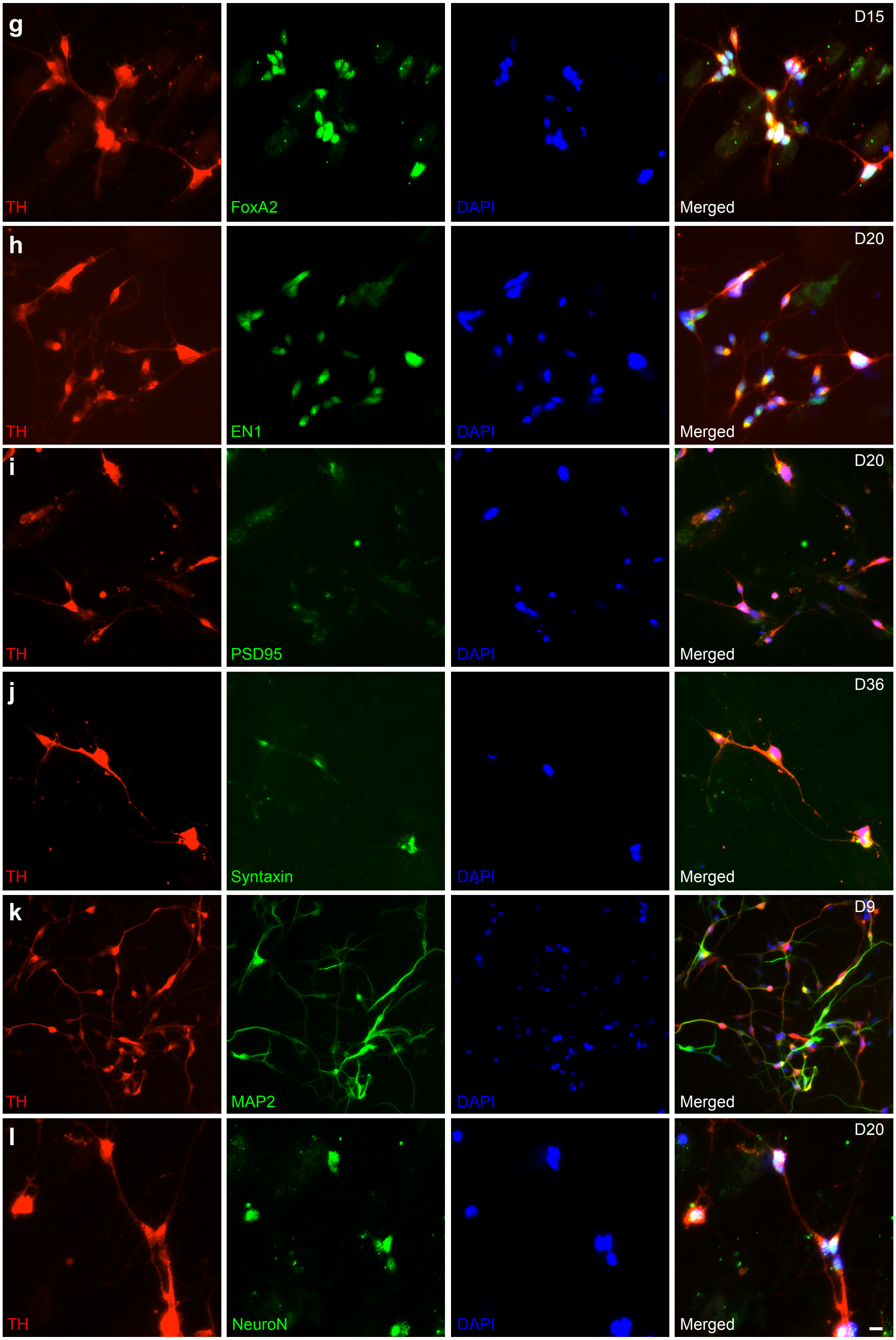

Supplementary Figure 13g-l. Separate channels of images in Fig. 7g-l. Bar, 10 μm

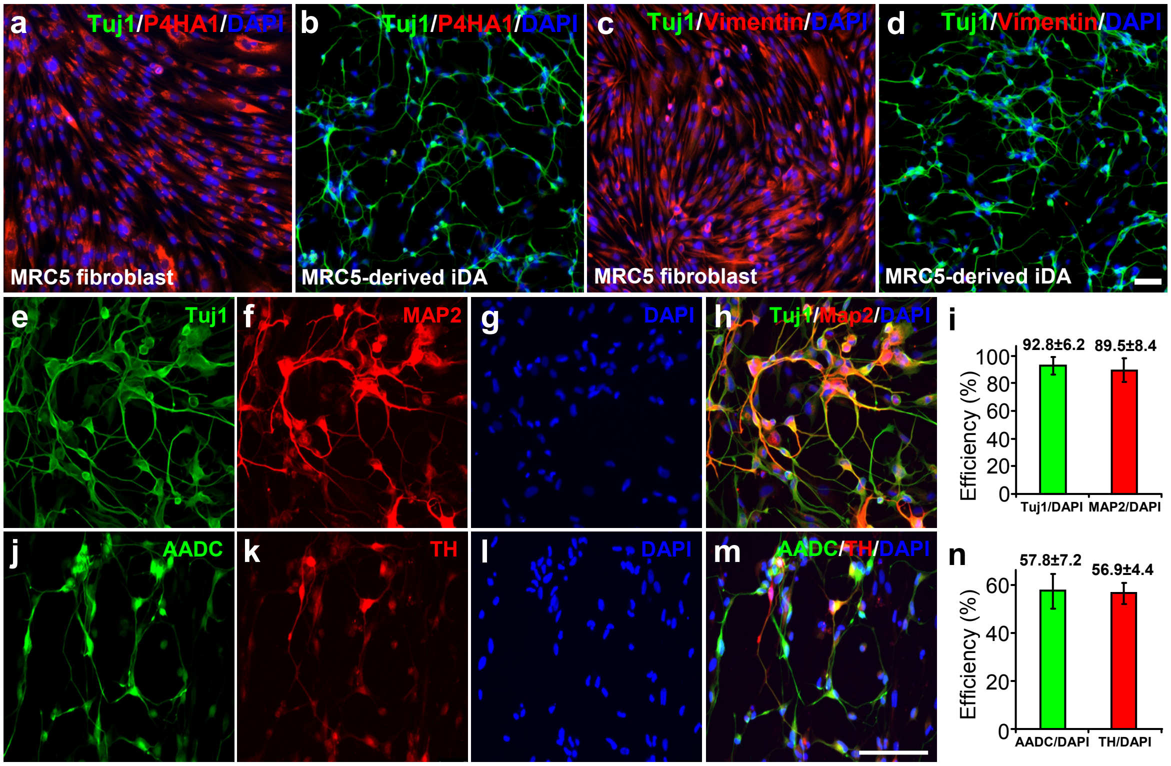

Supplementary Figure 14. Immunostaining and quantification of various markers in MRC5 cells and MRC5-derived iDA neurons. (**a-d**) MRC5 cells at day 0 (**a**, **c**) or MRC5-derived iDA neurons at day 10 (**b**, **d**) were co-stained for the fibroblast marker P4HA1 and the neuronal marker Tuj1 (**a**, **b**), or were co-stained for another fibroblast marker vimentin and the neuronal marker Tuj1 (**c**, **d**). (**e-i**) MRC5-derived iDA neurons at day 10 were co-stained for Tuj1 (**e**), MAP2 (**f**) and DAPI (**g**). Merged image (**h**) and quantification (**i**) showed that reprogramming efficiency for neurons as measured by the ratio of Tuj1/DAPI or MAP2/DAPI was not significantly different. (**j-n**) MRC5-derived iDA neurons at day 10 were co-stained for AADC (**j**), TH (**k**) and DAPI (**l**). Merged image (**m**) and quantification (**n**) showed that reprogramming efficiency for dopaminergic neurons as measured by the ratio of AADC/DAPI or TH/DAPI was not significantly different. Bars, 50 μm. Unpaired, two tailed Student's t-tests were used to calculate all *p* values.

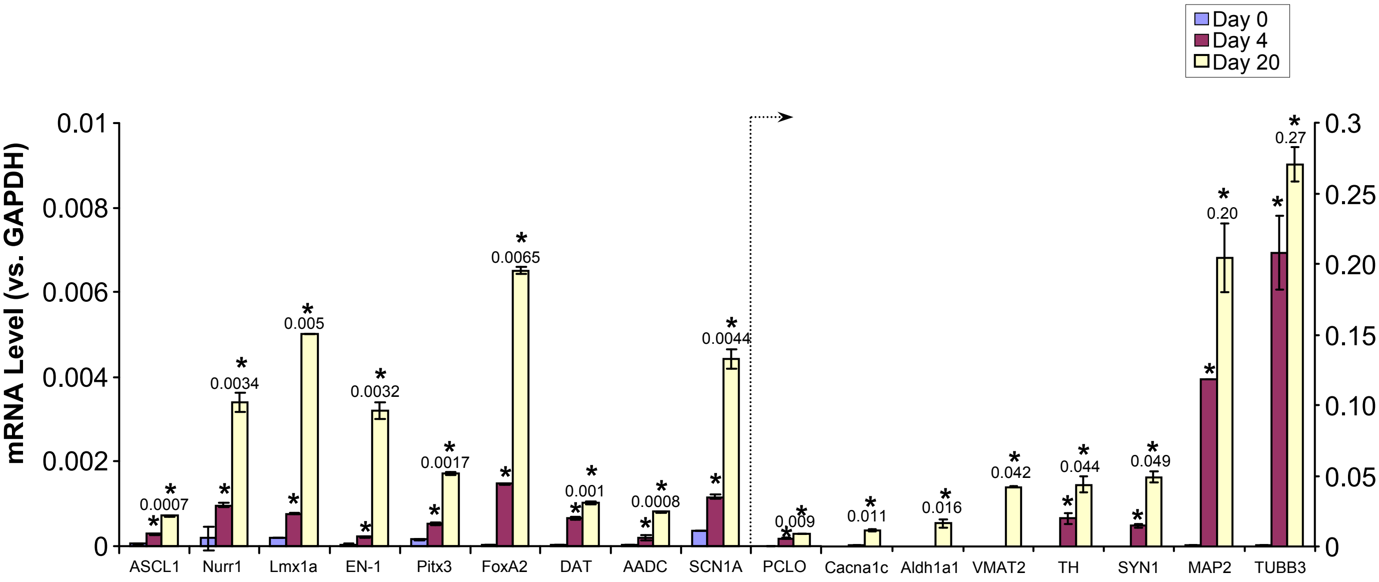

Supplementary Figure 15. The expression levels of endogenous genes during reprogramming. MRC5 cells were reprogrammed to iDA neurons using ANLmp and cell cycle arrest by serum withdrawal. Total mRNA was harvested at the indicated time points for qRT-PCR measurements of the indicated endogenous genes. Expression levels are relative to GAPDH levels. The levels of the last 8 genes are according to the right axis. *, $p < 0.01$ vs. day 0 for the same gene, n = 6 from 3 independent experiments. Unpaired, two tailed Student's t-tests were used to calculate all p values.

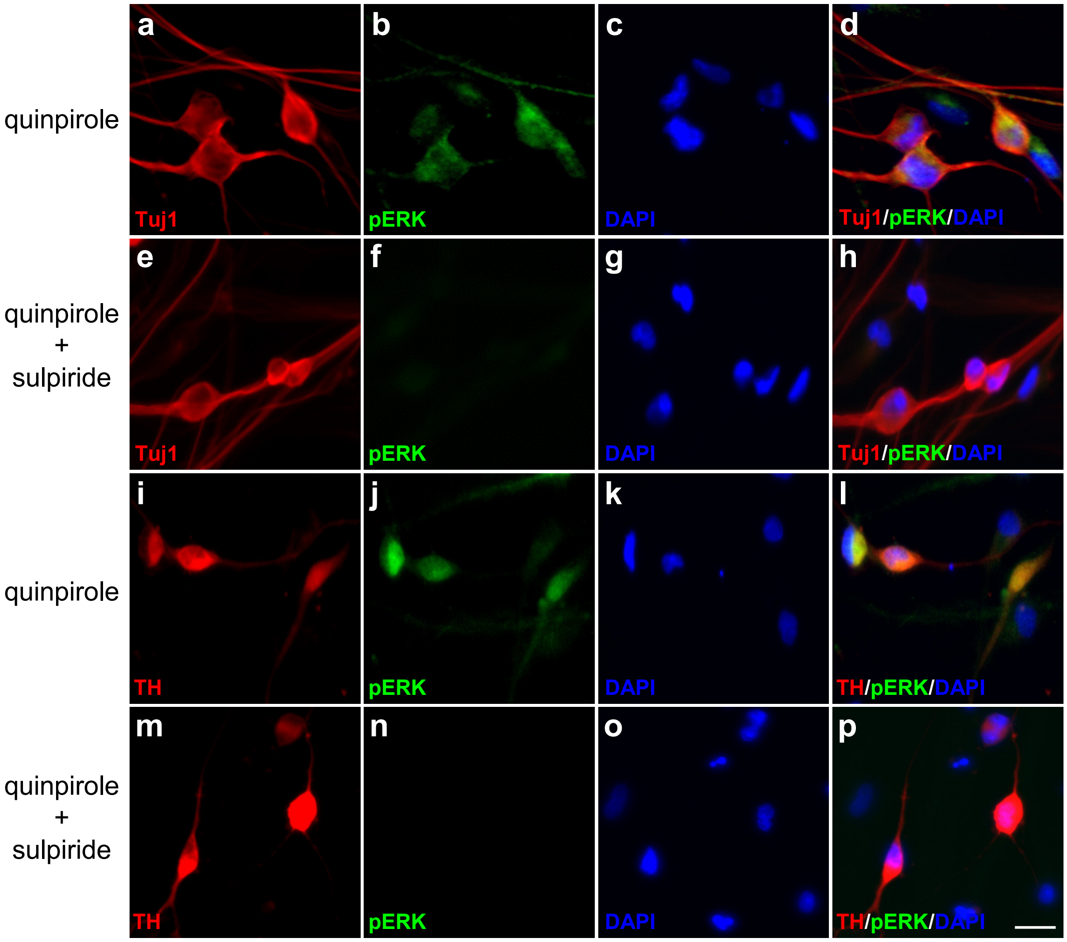

Supplementary Figure 16. Activation of ERK by dopamine D2-class agonist in iDA neurons. iDA neurons at day 15 were treated with the D2-class agonist quinpirole (1 μM for 10 min) (**a-d, i-l**) without or with the D2-class antagonist sulpiride (1 μM for 15 min, of which 5 min were before the addition of quinpirole) (**e-h, m-p**). The cultures were costained for Tuj1, pERK and DAPI (**a-h**) or TH, pERK and DAPI (**i-p**). Bar, 10 μm.

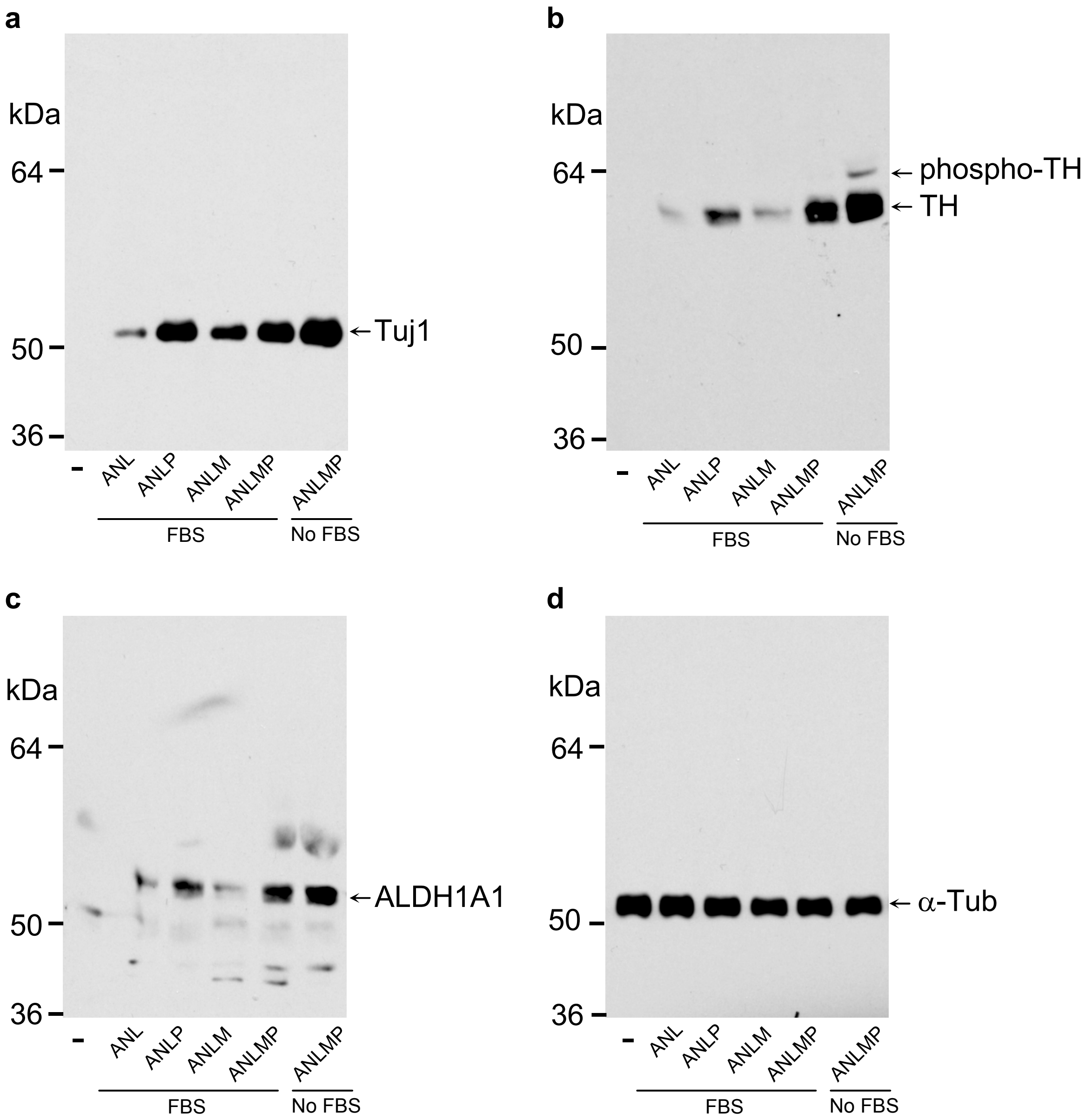

Supplementary Figure 17. Comparison of conversion efficiency by western blotting. MRC5 cells were infected without (-) or with the indicated combinations of lentiviruses and were reprogrammed with or without serum (FBS) during the first day of Dox induction. After another 9 days in Full Media, cells were lysed and blotted with antibodies against β3-tubulin (Tuj1) (**a**), TH (**b**), ALDH1A1 (**c**), or α-tubulin (as loading control) (**d**).

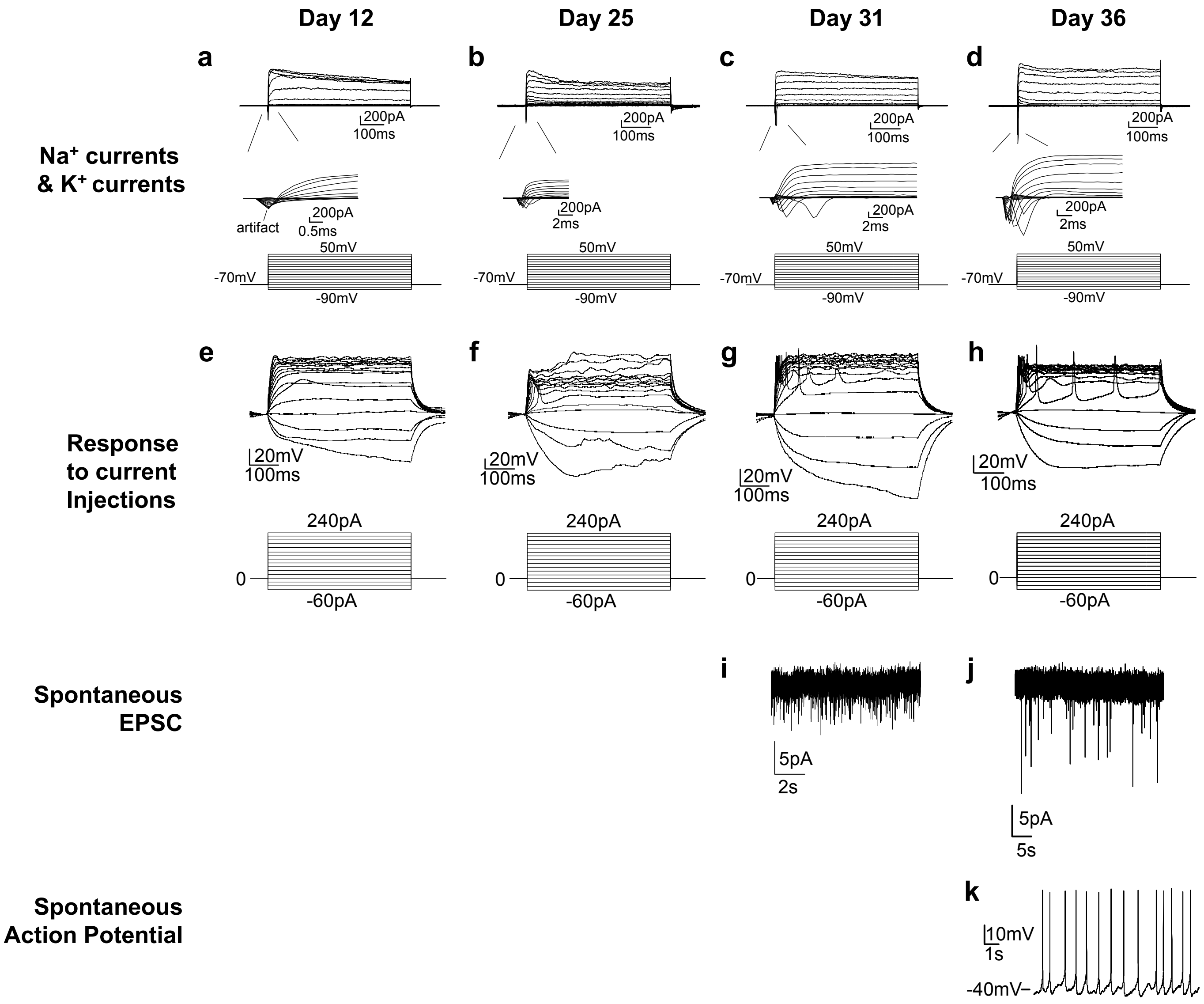

Supplementary Figure 18. Maturation of electrophysiological properties of iDA neurons. Electrophysiological recordings of iDA neurons at Day 12 (**a, e**), Day 25 (**b, f**), Day 31 (**c, g, i**) and Day 36 (**d, h, j, k**). Voltage dependent Na^+ currents and K^+ currents increased from undetectable (**a**) to significant levels (**b, c, d**). Response to current injections changed from no action potential (**e**) to increasingly mature action potentials (**f, g, h**). Spontaneous Excitatory Postsynaptic Currents (EPSCs) were observed at day 31 (**i**) and increased with time (**j**). Spontaneous action potentials were only detected at day 36 (**k**). Detailed quantifications of these electrophysiological parameters are in Supplementary Table 1.

Supplementary Table 1: Maturation of Electrophysiological Properties

	**Day 12**	**Day 25**	**Day 31**	**Day 36**
**Resting Membrane Potential** (mV)	-28.4 ± 2.8 (n=4)	-39.2 ± 1.7* (n=12)	-48.82 ± 3.8* (n=10)	-53.4±2.9* (n=8)
**Na^+ Currents (pA)** at -20mV	n.d.	352 ± 47 (n=15)	675 ± 86† (n=10)	1128±135† (n=8)
**K^+ Currents (pA)** at 50mV	n.d.	892 ± 56 (n=15)	1316±118‡ (n=10)	1576±126‡ (n=8)
**Evoked Action Potentials**	n.d.	7 out of 15 cells	10 out of 10 cells	8 out of 8 cells
**Spontaneous Action Potentials**	n.d.	n.d.	n.d.	3 out of 8 cells
**Spontaneous AP Frequency (Hz)**	n.d.	n.d.	n.d.	1.2 ± 0.3 (n=6)
**Spontaneous AP Amplitude (mV)**	n.d.	n.d.	n.d.	52.3 ± 5.4 (n=10)
**Spontaneous EPSC**	n.d.	n.d.	3 out of 10 cells	3 out of 8 cells
**Spontaneous EPSC frequency (Hz)**	n.d.	n.d.	5.1 ± 1.2 (n=8)	1.1 ± 0.2$^{\$}$ (n=12)
**Spontaneous EPSC amplitude (pA)**	n.d.	n.d.	4.4 ± 0.6 (n=9)	7.2 ± 0.5$^{\#}$ (n=10)

Data are presented as mean ± SEM. The number of neurons recorded from at least three independent experiments for each condition is included in each bracket. *, $p < 0.01$, vs. day 12 for resting membrane potential. †, $p < 0.05$, vs. day 25 for Na^+ current. ‡, $p < 0.05$, vs. day 25 for K^+ current. $^{\$}$, $p < 0.05$, vs. day 31 for spontaneous EPSC frequency. $^{\#}$, $p < 0.05$, vs. day 31 for spontaneous EPSC amplitude. Unpaired, two tailed Student's t-tests were performed to calculate all *p* values. n.d., not detected. AP, Action Potential. EPSC, Excitatory Postsynaptic Current.

Supplementary Table 2: Antibodies and their Dilutions

Protein	Species of Antibody	Vendor (catalog #)	Dilution
**p53**	Rabbit	Santa Cruz (sc-6243)	1:1000
**TH**	Rabbit	Thermo (OPA1-04050)	1:1000
**TH**	Mouse	Sigma(T2928)	1:500
**Tuj**	Mouse	Covance (MMS-435P-250)	1:1000
**En1**	Mouse	DSHB, Univ. Iowa (4G11-a)	1:200
**MAP2**	Mouse	Santa Cruz(sc-74421)	1:1000
**NeuroN**	Mouse	Millipore (MAB377)	1:500
**PSD95**	Rabbit	Cell Signaling (2507s)	1:1000
**Syntaxin-1**	Mouse	Life Technologies (P21943)	1:1000
**Nurr1**	Rabbit	Santa Cruz (sc-990)	1:1000
**FoxA2**	Rabbit	Abcam (ab40874)	1:1000
**Pitx3**	Rabbit	Life Technologies (382850)	1:1000
**AADC**	Rabbit	Millipore (AB1569)	1:2000
**DAT**	Rabbit	Millipore (AB1591p)	1:500
**VMAT2**	Rabbit	Millipore (AB1598p)	1:500
**ALDH1A1**	Rabbit	Abcam (ab23375)	1:2000
**5hmC**	Rabbit	Active Motif (39770)	1:2000
**P4HA1**	mouse	Millipore (MAB2701)	1:1000
**Vimentin**	mouse	Santa Cruz (SC-6260)	1:250
**Human NCAM**	mouse	Santa Cruz (SC-7326)	1:500
**synaptophysin**	rabbit	Thermo Scientific (PA1-1043)	1:1000
**AlexaFluor 488**	anti-Rabbit IgG	Life Technologies (A11008)	1:2000
**AlexaFluor 594**	anti-Rabbit IgG	Life Technologies (A11012)	1:2000
**AlexaFluor 488**	anti-mouse IgG	Life Technologies (A21202)	1:2000
**AlexaFluor 594**	anti-mouse IgG	Life Technologies (A11032)	1:2000
**AlexaFluor 594**	anti-mouse IgG2a	Life Technologies (A21135)	1:2000

Supplementary Table 3: Sequence of PCR Primers

Gene	Accession	Primer Sequences
**hASCL1** transgenic	NM_004316	Forward: TACTCGTCGGACGAGGGCTCTTA<br>Reverse: AAAGCAGCGTATCCACATAGCGTA
**hNURR1** transgenic	NM_006186	Forward: ACTTGGTGCCACCGCCAGCAATA<br>Reverse: AAAGCAGCGTATCCACATAGCGTA
**hLMX1a** transgenic	NM_001174069	Forward: AAACCCCATTGACCATCTGTACTC<br>Reverse: AAAGCAGCGTATCCACATAGCGTA
**hmiR-124** transgenic	MIMAT0000422	Forward: TCCCTCCGGCTGCCTGTC<br>Reverse: AAAGCAGCGTATCCACATAGCGTA
**hp53shRNA** transgenic	NM_000546	Forward: TACAGGGACAGCAGAGATCCACTT<br>Reverse: AACCGCAAGGAACCTTCCCGACTT
**hASCL1** endogenous	NM_004316	Forward: TACTCGTCGGACGAGGGCTCTTA<br>Reverse: GCACTAAAGATGCAGGTTGTGCGA
**hNURR1** endogenous	NM_006186	Forward: ACTTGGTGCCACCGCCAGCAATA<br>Reverse: GAGCTGAGACTGCTCACACGGCTA
**hLMX1a** endogenous	NM_001174069	Forward: AAACCCCATTGACCATCTGTACTC<br>Reverse: GGAAATGCTGAGCTACACCATATC
**hMAP2** endogenous	NM_031845	Forward: CAGGTGGCGGACGTGTGAAAATTGAGAGTG<br>Reverse: CACGCTGGATCTGCCTGGGGACTGTG
**hTUBB3** endogenous	NM_001197181	Forward: CGGTGGTGGAACCCCTACAAC<br>Reverse: AGGTGGTGACTCCGCTCAT
**hCACNA1C** endogenous	NM_001167625	Forward: TCCGCTGCTTCTGAAGATGA<br>Reverse: GGCCGTCGCTTTGGTAGTA
**hSCN1A** endogenous	NM_001165963	Forward: TGGGGAGTGGATAGAGACCA<br>Reverse: GAAAGAGATTCAGGACCACTAGG
**hSYN1** endogenous	NM_006950	Forward: TGAAGCCGGATTTTGTGCTGA<br>Reverse: GACCAAACTGCGGTAGTCTCC
**hPCLO** endogenous	NM_033026	Forward: CAGACACTTTCAGGTCAGAGC<br>Reverse: AGGCATCATACTAGACTTGTGCT
**hALDH1A1** endogenous	NM_000689.4	Forward: GAATTTCCCGTTGGTTATGCT<br>Reverse: TGTAGGCCCATAACCAGGAA
**hAADC** endogenous	NM_001082971	Forward: AGAACAGACTTAACGGGAGCCT<br>Reverse: CTGGACATGCTTGCGGATATAA
**hDAT** endogenous	NM_001044	Forward: AGCAGAACGGAGTGCAGCT<br>Reverse: GTATGCTCTGATGCCGTCT
**hVMAT2** endogenous	NM_003054	Forward: CTTTGGAGTTGGTTTTGC<br>Reverse: GCAGTTGTGATCCATGAG
**hGAPDH** endogenous	NM_002046.3	Forward: GACAACAGCCTCAAGATCATCAG<br>Reverse: ATGGCATGGACTGTGGTCATGAG
**hEn-1** endogenous	NM_001426	Forward: CCCTGGTTTCTCTGGGACTT<br>Reverse: GCAGTCTGTGGGGTCGTATT
**hPitx3** endogenous	NM_005029	Forward: GTGGGTGGAGAGGAGAACAA<br>Reverse: TTCCTCCCTCAGGAAACAATG
**hFOXA2** endogenous	NM_021784.4	Forward: CTGGGAGCGGTGAAGATGGA<br>Reverse: ACGTACGACGACATGTTCATGGAG
**hTH** endogenous	NM_000360	Forward: TCATCACCTGGTCACCAAGTT<br>Reverse: GGTCGCCGTGCCTGTACT

**hTet1** endogenous	NM_030625.2	Forward: CCGAATCAAGCGGAAGAATA<br>Reverse: ACTTCAGGTTGCACGGTCTC
**hTet2** endogenous	NM_001127208.2	Forward: AGCCCCATCACGTACAAAAC<br>Reverse: TGTGGTGGCTGCTTCTGTAG
**hTet3** endogenous	NM_144993.1	Forward: CAGCAGCCGAGAAGAAGAAG<br>Reverse: GGACAATCCACCCTTCAGAG
**hTP53** endogenous	NM_001126118.1	Forward: CACGAGCTGCCCCCAGG<br>Reverse: AGAATGTCAGTCTGAGT
