# Supplementary material for: Author Correction: Cell cycle and p53 gate the direct conversion of human fibroblasts to dopaminergic neurons
Source: Nat Commun. 2026 Oct 9;17:10654. doi: 10.1038/s41467-026-77919-w (PMC13652813; doi:10.1038/s41467-026-77919-w)

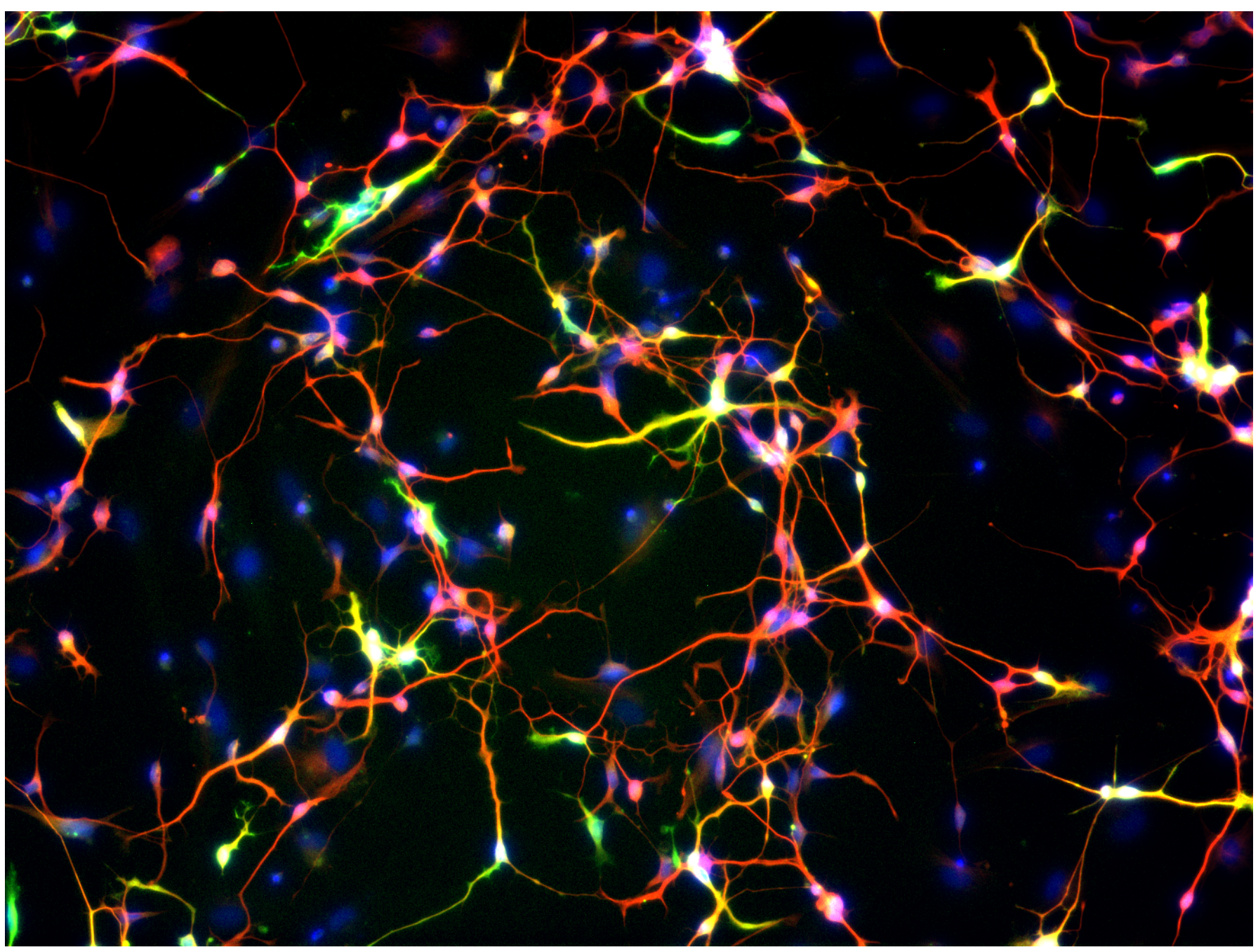

Supplement: Supplementary file 1 [file 41467_2026_77919_MOESM1_ESM.pdf]
